# A *Tetrahymena* Hsp90 co-chaperone promotes siRNA loading by ATP-dependent and ATP-independent mechanisms

**DOI:** 10.15252/embj.201490062

**Published:** 2015-01-14

**Authors:** Sophie L Woehrer, Lucia Aronica, Jan H Suhren, Clara Jana-Lui Busch, Tomoko Noto, Kazufumi Mochizuki

**Affiliations:** Institute of Molecular Biotechnology of the Austrian Academy of Sciences (IMBA)Vienna, Austria

**Keywords:** Argonaute protein, RISC assembly, RNAi, siRNA, *Tetrahymena*

## Abstract

The loading of small interfering RNAs (siRNAs) and microRNAs into Argonaute proteins is enhanced by Hsp90 and ATP in diverse eukaryotes. However, whether this loading also occurs independently of Hsp90 and ATP remains unclear. We show that the *Tetrahymena* Hsp90 co-chaperone Coi12p promotes siRNA loading into the Argonaute protein Twi1p in both ATP-dependent and ATP-independent manners *in vitro*. The ATP-dependent activity requires Hsp90 and the tetratricopeptide repeat (TPR) domain of Coi12p, whereas these factors are dispensable for the ATP-independent activity. Both activities facilitate siRNA loading by counteracting the Twi1p-binding protein Giw1p, which is important to specifically sort the 26- to 32-nt siRNAs to Twi1p. Although Coi12p lacking its TPR domain does not bind to Hsp90, it can partially restore the siRNA loading and DNA elimination defects of *COI12* knockout cells, suggesting that Hsp90- and ATP-independent loading of siRNA occurs *in vivo* and plays a physiological role in *Tetrahymena*.

## Introduction

Small RNA-mediated silencing pathways regulate a variety of cellular processes in eukaryotes (Dueck & Meister, 2014). Their effector complex, the RNA-induced silencing complex (RISC), consists of an Argonaute protein bound by an approximately 20- to 30-nt small RNA. Three major classes of Argonaute-bound small RNAs exist. MicroRNAs (miRNAs) and small interfering RNAs (siRNAs) are processed from double-stranded RNA precursors by the RNase III enzyme Dicer. They are loaded into the AGO subfamily of Argonaute proteins as an RNA duplex, and one of the two strands (the passenger strand) is then removed to form a mature RISC. In contrast, the third class of small RNAs, Piwi-interacting RNAs (piRNAs), are suggested to be produced from single-stranded precursor RNAs independently of Dicer and are loaded into the Piwi subfamily of Argonaute proteins.

*In vitro* RISC assembly assays using cell extracts have demonstrated that Hsp90 and Hsp90 co-chaperones enhance the loading of miRNAs and siRNAs into Argonaute proteins in flies and plants (Iwasaki *et al*, 2010; Iki *et al*, 2010, 2012; Miyoshi *et al*, 2010). *In vivo* genetic and pharmacological studies have also suggested that Hsp90 co-chaperones are required for the loading of miRNAs and siRNAs in mammals (Martinez *et al*, 2013; Pare *et al*, 2013). Therefore, Hsp90 most likely plays an important *in vivo* role in the loading of these double-stranded RNA-derived small RNAs. The chaperone function of Hsp90 is mediated through dynamic conformation changes that are induced by ATP binding and hydrolysis (Taipale *et al*, 2010). ATP hydrolysis by Hsp90 enhances the loading of miRNAs and siRNAs into human Ago2 (Yoda *et al*, 2010), fly Ago1 (Kawamata *et al*, 2009) and fly Ago2 (Miyoshi *et al*, 2010) in cell lysates, but it is dispensable for passenger strand removal for at least fly Ago1 (Kawamata *et al*, 2009). It has been suggested that an energy-consuming conformational opening allows Argonaute proteins to incorporate the bulky RNA duplex, and the following passenger strand removal is a passive process that is linked to the release of the structural tension caused by such an opening (Kawamata & Tomari, 2010). In contrast, in plants, the binding of ATP to Hsp90, but not its hydrolysis, enhances the loading of miRNAs and siRNAs into AGO1, whereas ATP hydrolysis by Hsp90 triggers the removal of the passenger strand from AGO1 (Iki *et al*, 2010). Therefore, ATP hydrolysis by Hsp90 plays different roles in different eukaryotes and/or different RISC assembly systems.

Hsp90 has also been implicated in the assembly of piRNA–Piwi protein complexes (piRISCs). The inhibition of Hsp90 in a silkworm culture cell line causes the reduction of piRNAs, accumulation of by-product short antisense RNAs, and inaccurate loading of precursor piRNAs (Xiol *et al*, 2012; Izumi *et al*, 2013). In addition, a loss of the putative Hsp90 co-chaperone Shutdown causes defects in the accumulation and loading of piRNAs in *Drosophila* (Olivieri *et al*, 2012; Preall *et al*, 2012). Similarly, the mouse Shutdown homolog FKBP6 is required for the biogenesis of Miwi2-associated piRNAs (Xiol *et al*, 2012). These results indicate that Hsp90 and Hsp90 co-chaperones function in both the loading of primary piRNAs and the endonucleolytic “ping-pong” amplification cycle of secondary piRNAs.

Although it is widely accepted that Hsp90 enhances the loading process of small RNAs into Argonaute proteins in an ATP-dependent manner in a variety of eukaryotes, whether Hsp90- and ATP-independent RISC assembly pathways occur *in vivo* remains unclear. Although the presence of ATP enhances the loading of miRNAs and siRNAs to human Ago2 in cell lysates (Yoda *et al*, 2010), these RNAs are also loaded, albeit to a lesser extent, into human Ago2 without ATP in the cell lysate and in reconstituted systems with immunopurified or recombinantly expressed human Ago2 *in vitro* (Gregory *et al*, 2005; Maniataki & Mourelatos, 2005; MacRae *et al*, 2008; Yoda *et al*, 2010). A previous study suggested that this ATP-independent small RNA loading is a bypass mechanism by which spontaneously formed single-stranded RNAs from siRNA and miRNA duplexes are incorporated into the Argonaute protein (Yoda *et al*, 2010). However, whether such ATP-independent loading is solely mediated by single-stranded RNAs and whether it constitutes a functional loading mechanism *in vivo* remain unknown.

The ciliated protozoan *Tetrahymena thermophila* has at least two siRNA pathways. The first pathway utilizes approximately 23- to 24-nt siRNAs, which are constitutively expressed, processed by the Dicer protein Dcr2p (Lee & Collins, 2007) and loaded into the Argonaute protein Twi2p (Lee & Collins, 2006, 2007; Couvillion *et al*, 2009). The second pathway consists of approximately 26- to 32-nt siRNAs, named scan RNAs (scnRNAs), which are expressed exclusively during sexual reproduction (called conjugation), are processed by the Dicer protein Dcl1p (Malone *et al*, 2005; Mochizuki & Gorovsky, 2005), and are loaded into the Argonaute protein Twi1p (Mochizuki *et al*, 2002; Mochizuki & Gorovsky, 2004).

**Figure 1:**
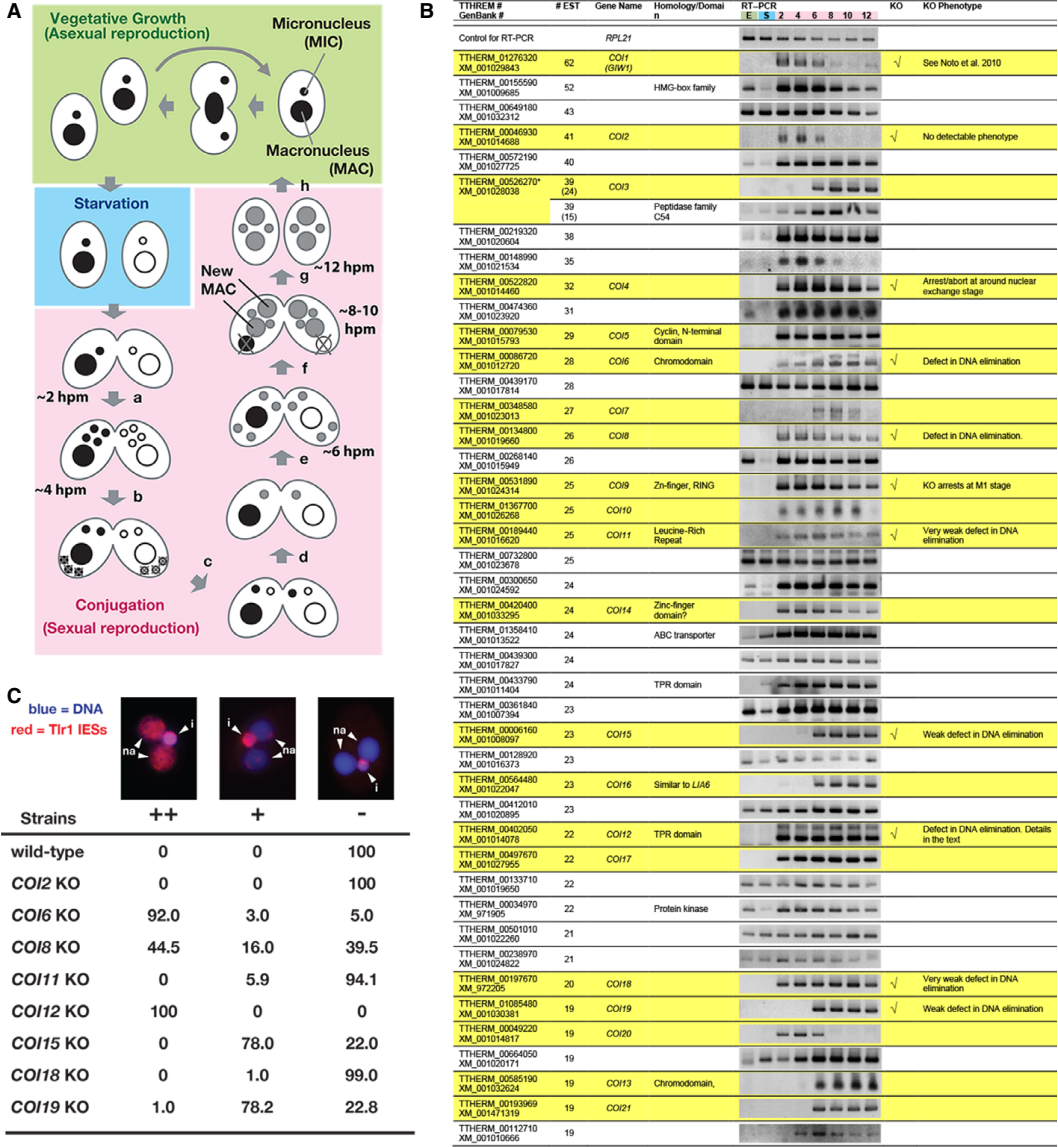
Identification and characterization of *COI* genes

Although the biological role of the constitutively expressed 23- to 24-nt siRNAs is not clear, the scnRNA pathway plays an important role in programmed DNA elimination in *Tetrahymena* (Chalker *et al*, 2013), which occurs during conjugation when the germline micronucleus (MIC) differentiates into the somatic macronucleus (MAC; Fig1A). Programmed DNA elimination removes approximately 1/3 of the germline genome, which is distributed throughout the MIC genome as > 8,000 internal eliminated sequences (IESs), from the newly formed MAC (Fass *et al*, 2011; Schoeberl *et al*, 2012). During conjugation, scnRNA duplexes are produced in the MIC by Dcl1p (Malone *et al*, 2005; Mochizuki & Gorovsky, 2005) and are exported to the cytoplasm, where they are loaded into Twi1p to form a pre-scnRISC (a complex of Twi1p and a scnRNA duplex; Mochizuki & Gorovsky, 2004; Noto *et al*, 2010). The endoribonuclease (“Slicer”) activity of Twi1p cleaves the passenger strand of the scnRNA duplex to facilitate the formation of a mature scnRISC (Twi1p-guide scnRNA complex; Noto *et al*, 2010). The Twi1p-binding protein Giw1p interacts with mature scnRISCs but not with pre-scnRISCs and selectively imports mature scnRISCs to the parental MAC (Noto *et al*, 2010) where scnRNAs that are complementary to the MAC genome (= non-IESs) are degraded (Mochizuki & Gorovsky, 2004; Schoeberl *et al*, 2012). The remaining scnRISCs containing IES-complementary scnRNAs are then moved into the new MAC and guide the heterochromatin-mediated excision of IESs (Taverna *et al*, 2002; Liu *et al*, 2007; Vogt & Mochizuki, 2013).

Life cycle of *Tetrahymena*. A single cell of *Tetrahymena thermophila* contains two different types of nuclei: a macronucleus (MAC) and a micronucleus (MIC). When sufficient nutrients are available, *Tetrahymena* grows by binary fission, and the MAC and MIC are divided independently (vegetative growth, green). After prolonged starvation (blue), two cells of complementary mating types fuse to begin the sexual reproduction process (conjugation, pink). Their MICs undergo meiosis (a), and one of the meiotic products survives and divides mitotically, giving rise to two pronuclei: one stationary and one migratory (b). The migratory gametic nucleus crosses the conjugation bridge (c) and fuses with the stationary nucleus to produce the zygotic nucleus (d). The zygotic nucleus divides twice (e). Two of the products differentiate into the new MACs, whereas the other two remain as MICs (f). The parental MAC is degraded, and the pair is dissolved (g). The exconjugants resume vegetative growth when nutrients are available (h). The approximate time at which each event occurs in our culture condition is indicated (hpm: hours post-mixing).

The expression of the predicted *Tetrahymena* genes showing more than 19 expressed sequence tags (ESTs) from conjugating cells but not vegetative cells was analyzed by RT–PCR using total RNAs from exponentially growing vegetative cells (“E”); 16-h starved cells (“S”); or conjugating cells at 2, 4, 6, 8, 10, and 12 hpm. Twenty-two genes that were exclusively expressed during conjugation are marked with a yellow background. Eleven genes that were analyzed by gene knockout (KO) are indicated in the “KO” column. The phenotypes are described in the “KO phenotype” column. The predicted TTHERM_00526270 contains two genes, which were analyzed separately in this study.

Exconjugants of wild-type (WT) and KO strains for the indicated *COI* genes at 36–48 hpm were used to detect Tlr1 IES elements by fluorescence in situ hybridization (FISH). The percentages of total exconjugants that showed severe (++), mild (+), and no (−) DNA elimination defects are given (*n *>* *100). Representative pictures of exconjugants showing each phenotype are shown above. The Tlr1 FISH signal is in red, and the DNA was stained with DAPI (blue). The MICs (i) and the new MACs (na) are marked.

How scnRNAs are loaded into Twi1p and what mechanisms sort 26- to 32-nt scnRNAs into Twi1p are poorly understood. Here, we report an *in vitro* and *in vivo* functional analysis of the Hsp90 co-chaperone Coi12p and uncover the existence of both ATP-dependent and ATP-independent loading mechanisms of the scnRNA duplex into the Argonaute protein Twi1p, which are coupled with the sorting of scnRNAs.

## Results

### Identification and functional analyses of *Conjugation-induced (COI)* genes

Most genes that are required for DNA elimination are expressed exclusively during conjugation, the sexual reproduction process (Fig1A) of *Tetrahymena* (Chalker *et al*, 2013). Therefore, to identify novel genes involved in DNA elimination, we aimed to explore genes that are specifically expressed during conjugation. We first chose 44 previously uncharacterized genes showing expressed sequence tags (ESTs) only from conjugating cells but not from vegetative cells (listed in Fig1B). Then, the expression of these genes was analyzed by RT–PCR using total RNA from exponentially growing vegetative cells (“E” in Fig1B); 16-h starved cells (“S” in Fig1B); or conjugating cells at 2, 4, 6, 8, 10, and 12 h post-mixing (hpm; corresponding numbers in Fig1B). For 22 of the genes, we detected PCR products exclusively from conjugating cells (Fig1B, marked with yellow). A microarray-based RNA expression analysis (Miao *et al*, 2009) also supports their conjugation-specific expression (Supplementary Fig S1). We named these 22 genes *Conjugation-induced* (*COI*) genes.

We produced gene knockout (KO) strains for 11 *COI* genes (in the “KO” column in Fig1B, see also Supplementary Fig S2). *COI1* encodes the Twi1p-interacting protein Giw1p, for which we previously reported detailed analyses (Noto *et al*, 2010). The KO strains for *COI4* and *COI9* aborted conjugation during or directly after meiosis (data not shown). We did not analyze these genes further in this study. For KO strains of the other 8 *COI* genes, we analyzed their DNA elimination by fluorescence in situ hybridization (FISH) using probes against *Tlr1* IESs (Fig1C), which are moderately repeated (∽30 copies) transposon-related sequences in the MIC genome (Wuitschick *et al*, 2002). In the exconjugants (sexual progeny) of wild-type cells, *Tlr1* IESs were detected only in the MIC (Fig1C, “i”) but not in the new MACs (Fig1C, “na”), reflecting the successful DNA elimination of IESs from the new MACs (Fig1C, “−”). In the KO strains for *COI6* and *COI12, Tlr1* IESs were detected homogeneously (Fig1C, “++”) in most of the new MACs. In the *COI8* KO strains*, Tlr1* IESs were detected homogeneously in some of the new MACs, whereas they were partially (Fig1C, “+”) or completely removed from the other new MACs. In the KO strains for *COI11*,*COI15, COI18,* and *COI19,* punctate staining for *Tlr1* IESs was detected in a majority (*COI15* and *COI19* KOs) or some (*COI11 and COI18* KOs) of the new MACs, whereas they were absent in the other cells (Fig1C). These results indicate that *COI6* and *COI12* are essential for DNA elimination and that *COI8*,*COI11, COI15*,*COI18,* and *COI19* play some important, although not essential, role in DNA elimination. In this study, we report further analyses of *COI12*.

### Coi12p is expressed exclusively during conjugation and localizes to both the cytoplasm and MACs

**Figure 2:**
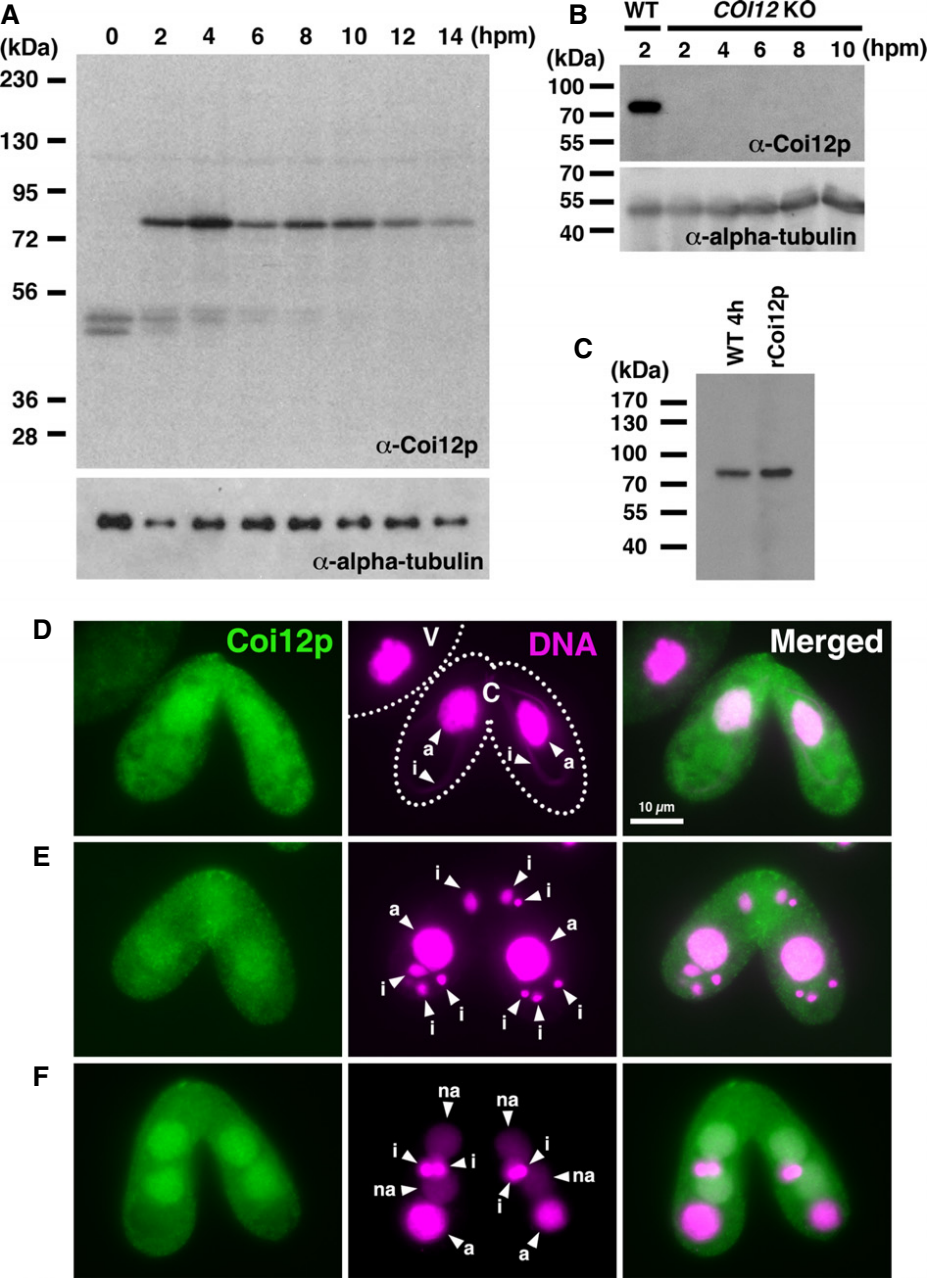
Expression and localization of Coi12p

To analyze the localization of Coi12p, the protein encoded by *COI12*, we raised an antibody against recombinant Coi12p expressed in *E. coli*. Based on Western blotting, this antibody recognized a *Tetrahymena* protein, which appeared from early to late conjugation stages (Fig2A, 2–14 hpm) but not in starved vegetative cells (Fig2A, 0 hpm). This pattern is consistent with the *COI12* mRNA expression pattern detected by RT–PCR (Fig1B). The protein recognized by the antibody was not detected in *COI12* KO cells (Fig2B). Although the predicted molecular weight of Coi12p is 64.3 kDa, the protein recognized by the antibody migrated as an approximate 75-kDa protein by SDS–PAGE. Although the reason for this difference is not clear, we found that the recombinant Coi12p expressed in *E. coli* also migrated as an approximate 75-kDa protein (Fig2C). Together, these results indicate that the antibody specifically recognizes Coi12p.

Proteins extracted from wild-type cells at the indicated time point of conjugation (hpm) were analyzed by Western blot with the newly established anti-Coi12p antibody (top) and with an anti-alpha-tubulin antibody (bottom). The positions of the protein molecular weight markers are indicated at the left.

Proteins extracted from wild-type cells at 2 hpm and from the *COI12*KO cells at the indicated time of conjugation (hpm) were analyzed by Western blot as described in (A).

Proteins extracted from wild-type cells at 4 hpm and recombinantly expressed Coi12p from *E. coli* were analyzed by Western blot as described in (A).

Immunofluorescence staining of wild-type cells using the anti-Coi12p antibody. Coi12p localizes to the cytoplasm and the MAC in early stage (D, meiotic prophase), mid-stage (E, nuclear exchange), and late stage (F, nuclear alignment) of conjugation. The MICs (i), parental MACs (a) and newly developed MACs (na) are marked with arrowheads. Vegetative (V) and conjugating (C) cells are circled with dotted lines in (D).

We next analyzed the localization of Coi12p by indirect immunofluorescence staining using the anti-Coi12p antibody. Consistent with the Western blot result above (Fig2A), Coi12p was detected only in conjugating cells (Fig2D marked with “C”) but not in vegetative cells (marked with “V”). Coi12p was detected in both the cytoplasm and the parental and new MACs (marked “a” and “na”, respectively) throughout conjugation (Fig2D–F).

### *COI12* is required for parental MAC localization and the stable accumulation of Twi1p

**Figure 3:**
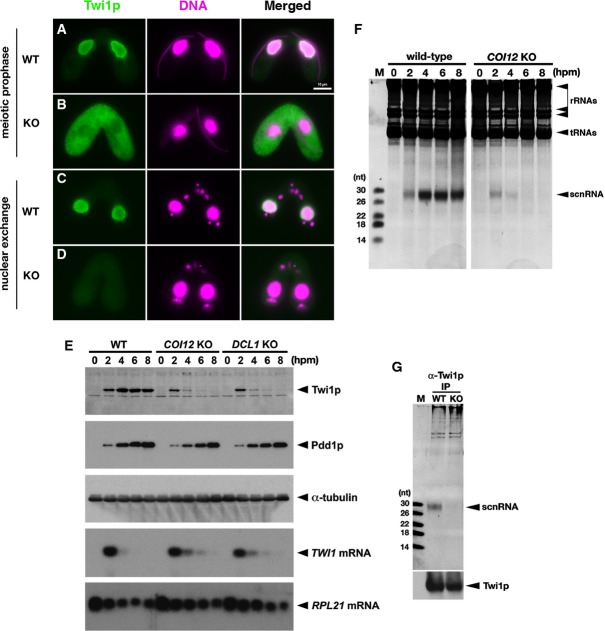
Coi12p is necessary for the loading of scnRNAs into Twi1p *in vivo*

Because *COI12* is required for DNA elimination (Fig1C) and the Argonaute protein Twi1p plays a pivotal role in DNA elimination (Mochizuki *et al*, 2002; Mochizuki & Gorovsky, 2004), we asked whether the localization of Twi1p was altered in the absence of *COI12*. Immunofluorescence staining using an anti-Twi1p antibody (Fig3) showed that in wild-type cells, Twi1p localized in the parental MACs at the early stages of conjugation (∽2–4 hpm, Fig3A). In contrast, Twi1p was mainly detected in the cytoplasm in the early stages of conjugation in *COI12* KO cells (Fig3B), indicating that *COI12* is essential for the MAC localization of Twi1p.

The localization of Twi1p in wild-type (A, C) and *COI12*KO (B, D) cells at the early (meiotic prophase; A, B) and mid- (nuclear exchange; C, D) conjugation stages was analyzed by immunofluorescence staining using an anti-Twi1p antibody. Twi1p was localized in the parental MACs at both stages in wild-type cells (A, C). In contrast, in *COI12*KO cells, Twi1p was localized in the cytoplasm at the early stage (B) and became undetectable at the mid-stage (D). All pictures share the scale bar (10 μm) shown in (A).

The expression of Twi1p in wild-type, *COI12*KO, and *DCL1*KO cells at the indicated time points of conjugation was analyzed by Western blot using an anti-Twi1p antibody (top panel). The expression of Pdd1p was analyzed using an anti-Pdd1p antibody as an indicator of the proper progress of conjugation (2^nd^ panel), and the expression of alpha-tubulin was analyzed by anti-alpha-tubulin antibody as a loading control (3^rd^ panel). The expression of *TWI1*mRNA (4^th^ panel) and *RPL21*mRNA (bottom panel) was analyzed by Northern blot.

Total RNA was extracted from wild-type and *COI12*KO cells at the indicated time points of conjugation, separated on a denaturing gel, and visualized by nucleic acid staining (GelRed). Bands corresponding to rRNAs, tRNAs, and scnRNAs are marked with an arrowhead.

Twi1p was immunoprecipitated with an anti-Twi1p antibody from wild-type (WT) and *COI12*KO (KO) cells at 2 hpm. Co-precipitated RNAs were separated on a denaturing gel and visualized by the nucleic acid dye GelRed. The position of scnRNAs is marked with an arrowhead. The sizes of RNA markers (M) are indicated at the left. Precipitated Twi1p was analyzed by Western blot using an anti-Twi1p antibody (bottom).

In wild-type cells, Twi1p remained in the parental MACs at the mid-conjugation stages (∽4–6 hpm, Fig3C). In contrast, in the *COI12* KO strains, Twi1p was hardly detected at the mid-conjugation stages (Fig3D). This result was also confirmed by Western blotting (Fig3E); although a comparable amount of Twi1p was detected at 2 hpm in both wild-type and *COI12* KO cells, the amount of Twi1p at 4 hpm was much lower in *COI12* KO cells than in wild-type cells, and Twi1p became undetectable in *COI12* KO cells at later stages. A Northern hybridization analysis revealed that *TWI1* mRNA predominantly accumulates at an early conjugation stage (2 hpm) in wild-type cells (Fig3E), indicating that Twi1p is translated at the early conjugation stage and stably persists in the later stages in wild-type cells. Because similar levels of *TWI1* mRNA were detected in wild-type and *COI12* KO cells (Fig3E) and the initial production of Twi1p is unaffected in *COI12* KO cells (Fig3E), we conclude that Twi1p is destabilized in the absence of *COI12*.

### *COI12* is required for the loading of scnRNAs into Twi1p

The Dicer protein Dcl1p, which is required for the production of scnRNAs (Malone *et al*, 2005; Mochizuki & Gorovsky, 2005), is also required for the parental MAC localization of Twi1p (Noto *et al*, 2010). Furthermore, similar to Coi12p, we found that Dcl1p is required for the stable accumulation of Twi1p, without affecting the *TWI1* mRNA level (Fig3E). Therefore, we hypothesized that Coi12p could also be required for the biogenesis of scnRNAs. However, we detected a comparable amount of scnRNAs in wild-type and *COI12* KO cells at the early conjugation stage (2 hpm), although scnRNAs became undetectable in mid- and late conjugation stages (4–8 hpm) in *COI12* KO cells (Fig3F). Thus, we conclude that Coi12p is not required for the production of scnRNAs but is required for their stable accumulation.

The formation of the scnRNA–Twi1p complex (scnRISC) is essential for the stable accumulation of both scnRNAs and Twi1p and for the parental MAC localization of Twi1p (Mochizuki & Gorovsky, 2004; Noto *et al*, 2010). Therefore, we next examined whether Twi1p is loaded with scnRNA in *COI12* KO cells. Twi1p was immunoprecipitated from wild-type and *COI12* KO cells at 2 hpm using an anti-Twi1p antibody, and the co-precipitated RNA was analyzed. Notably, although Twi1p and scnRNAs are unstable in the absence of *COI12*, there was no notable difference in the expression of Twi1p and scnRNA between wild-type and *COI12* KO cells at 2 hpm (Fig3E and F), and a similar amount of Twi1p was precipitated from both strains at this time point (Fig3G, bottom). As shown in Fig3G, scnRNAs were co-precipitated with Twi1p from wild-type cells, whereas no detectable scnRNA was precipitated with Twi1p from *COI12* KO cells (Fig3G, top). These results indicate that *COI12* is required at a step downstream of the production of scnRNAs and upstream of or directly at the formation of scnRISC.

We previously demonstrated that the MAC localization of Twi1p is dependent on its loading with scnRNAs and is required for DNA elimination (Noto *et al*, 2010). Therefore, all of the phenotypes of *COI12* KO cells described above, including the defect in DNA elimination (Fig1B), the cytoplasmic mis-localization of Twi1p (Fig3B), and the instabilities of Twi1p and scnRNAs (Fig3D–F), can be explained by a defect in the loading of scnRNAs into Twi1p in the absence of Coi12p. We conclude that the primary function of Coi12p in the DNA elimination pathway is to promote the loading of scnRNAs into Twi1p.

### Coi12p acts directly in scnRNA loading into Twi1p

**Figure 4:**
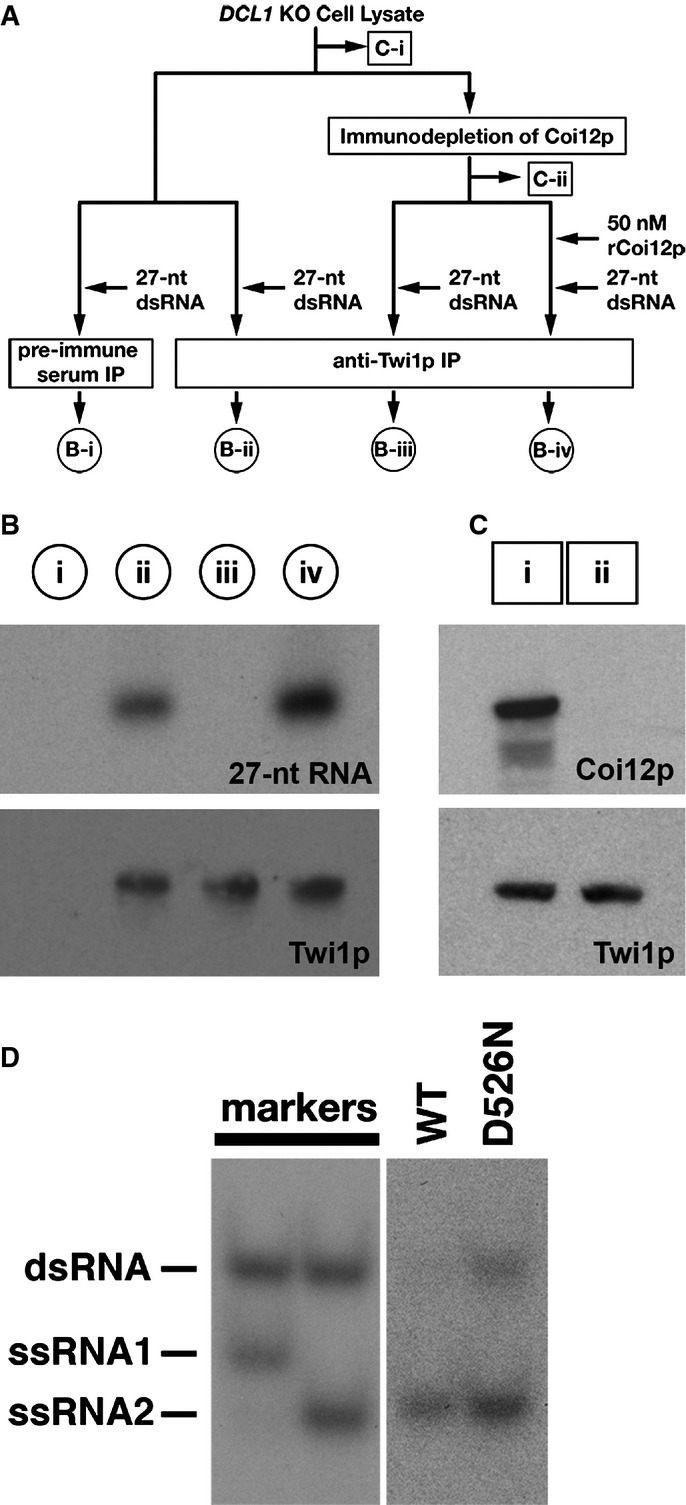
Coi12p is necessary for the loading of scnRNAs to Twi1p in cell lysates

To better understand the role of Coi12p, we established a cell lysate-based scnRNA loading assay (Fig4A). We synthesized a 27-nt RNA duplex, which was designed from a sequenced endogenous scnRNA (Lee & Collins, 2006). The duplex possesses 2-nt 3′-overhangs that mimic Dcl1p-processed scnRNAs (Mochizuki & Kurth, 2013), and both strands were radiolabeled at their 5′ ends. Lysates from mating *DCL1* KO cells, which lack endogenous scnRNAs (Malone *et al*, 2005; Mochizuki & Gorovsky, 2005), were incubated with the radiolabeled 27-nt RNA duplex, Twi1p was precipitated with an anti-Twi1p antibody, and co-precipitated RNA was analyzed by autoradiography. The labeled RNA was co-precipitated with an anti-Twi1p antibody (Fig4B, lane ii) but not with pre-immune serum (Fig4B, lane i), indicating that scnRNA duplexes can be loaded into Twi1p in *Tetrahymena* cell lysates.

A schematic drawing of the scnRNA loading assays in cell lysates. The circled samples (B-i, B-ii, B-iii, and B-iv) correspond to the lanes shown in (B). The boxed samples (C-i, C-ii) correspond to the lanes shown in (C). See text for details.

The 27-nt RNAs that co-precipitated with Twi1p from lysates with the different conditions shown in (A) were separated on a denaturing gel and detected by autoradiography (top). Precipitated Twi1p was detected by Western blot using an anti-Twi1p antibody (bottom).

Proteins in the cell lysate before (i) and after (ii) immunodepletion of Coi12p were analyzed by Western blot using anti-Coi12p (top) and anti-Twi1p (bottom) antibodies.

Cell lysate from the cells expressing FLAG-HA-tagged wild-type Twi1p (WT) or Twi1p in which the aspartic acid 526 was replaced with asparagine (D526N) at 3 hpm was incubated with radiolabeled double-stranded 27-nt RNAs. The Twi1p-containing complex was immunoprecipitated with an anti-Twi1p antibody, co-precipitated RNAs were separated on a native gel, and 27-nt RNAs were detected by autoradiography. As markers, ssRNA1 and ssRNA2, which were used for producing the radiolabeled double-stranded 27-nt RNAs, were mixed in 2:1 (left) or 1:2 (right) ratio, allowed to form double-stranded RNAs, and were separated in the same native gel.

Next, to assess the importance of Coi12p in the loading process, Coi12p was immunodepleted from the *DCL1* KO cell lysate with the anti-Coi12p antibody. The complete removal of Coi12p was confirmed by Western blotting (Fig4C). Then, the Coi12p-depleted lysate was incubated with the radiolabeled 27-nt RNA duplex in the presence or absence of bacterially expressed recombinant Coi12p, which was approximately 10-fold more concentrated (50 nM) than endogenous Coi12p in the original *DCL1* KO cell lysate (Supplementary Fig S3). Finally, Twi1p was precipitated with an anti-Twi1p antibody, and the co-precipitated RNA was analyzed. The depletion of Coi12p completely inhibited the co-precipitation of the labeled 27-nt RNAs with Twi1p (Fig4B, lane iii), and the addition of recombinant Coi12p restored the co-precipitation of Twi1p and 27-nt RNAs (Fig4B, lane iv). These results indicate that Coi12p must be present simultaneously with Twi1p and a scnRNA duplex to promote scnRISC formation in a cell lysate.

The scnRNA loading assays above detected the interaction between Twi1p and the 27-nt RNAs but did not show that these RNAs were properly loaded into Twi1p. To test the proper loading of RNAs in our loading assay system, we determined whether the 27-nt RNAs co-precipitated with Twi1p were single-stranded. This is based on the fact that in wild-type cells, Twi1p has an endoribonuclease (Slicer) activity that cuts one of the two strands (the passenger strand) of loaded scnRNAs, and this activity is required for efficiently removing the passenger strand from Twi1p (Noto *et al*, 2010). Because loaded scnRNAs must be appropriately placed in Twi1p for the endoribonucleolytic cleavage, we believe the double- to single-strand conversion of RNAs is a strong indication for the proper loading of RNAs into Twi1p. Lysates from mating cells expressing FLAG-HA-tagged wild-type Twi1p or Twi1p lacking Slicer activity because of a point mutation (D526N) were incubated with the radiolabeled 27-nt RNA duplex. Twi1p was then precipitated with an anti-Twi1p antibody, and co-precipitated RNA was analyzed by native gel electrophoresis (Fig4D). We found that RNAs co-precipitated with the wild-type Twi1p were mostly single-stranded (Fig4D, WT), whereas those that co-precipitated with Twi1p lacking the Slicer activity partially remained double-stranded (Fig4D, D526N). These results suggest that RNAs co-precipitated with Twi1p in the cell lysate-based scnRNA loading assays were properly loaded into Twi1p.

### FKBDs, but not the TPR domain, of Coi12p are essential for its scnRNA loading-promoting activity in cell lysates

**Figure 5:**
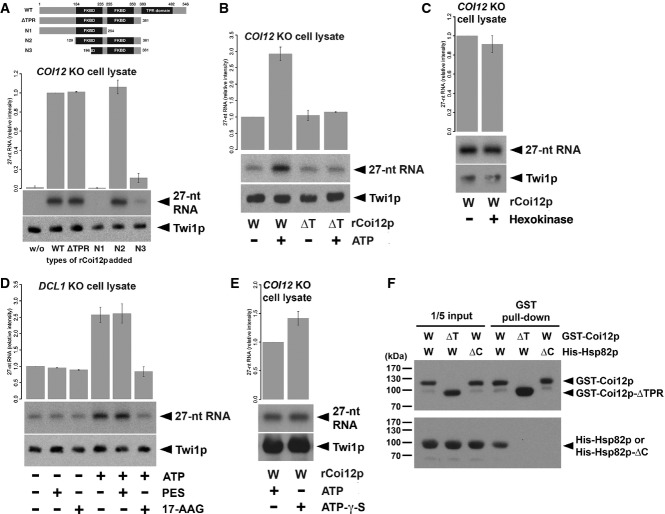
The TPR domain of Coi12p and Hsp90 are required for the enhancement of scnRNA loading by ATP in cell lysates

Coi12p has two FK506-binding domains (FKBDs) and a tetratricopeptide repeat (TPR) domain (Fig5A and Supplementary Fig S4). This domain architecture is common in many co-chaperones (Pearl & Prodromou, 2006). The FKBDs of some FK506-binding proteins (FKBPs) have peptidyl-prolyl cis-trans isomerase (PPIase) activity. Consistent with the rather poor sequence conservation between the FKBDs of Coi12p and active PPIases (Supplementary Fig S4A), we failed to detect PPIase activity from recombinant Coi12p (Supplementary Fig S5). Therefore, although we cannot exclude the possibility that Coi12p has a PPIase activity only for some specific substrates, the FKBDs of Coi12p most likely lack PPIase activity and may instead play a role in protein–protein interactions, similar to many other PPIase-inactive FKBDs (Galat, 2008; Rohl *et al*, 2013). The TPR domain is a common module for interaction between co-chaperones and the Hsp70 and/or Hsp90 chaperones (Haslbeck *et al*, 2013).

Cell lysate from *COI12*KO cells at 3 hpm was incubated with radiolabeled double-stranded 27-nt RNAs and the different truncated mutants of Coi12p (schematically shown on the top). The Twi1p-containing complex was immunoprecipitated with an anti-Twi1p antibody, co-precipitated RNAs were separated on a denaturing gel, and 27-nt RNAs were detected by autoradiography. The intensities of the bands in the different conditions relative to the wild-type (WT) sample (2^nd^ column) are shown on the top. A representative experiment is shown on the bottom.

Cell lysate from *COI12*KO cells at 3 hpm was incubated with radiolabeled double-stranded 27-nt RNAs, wild-type (W), or ΔTPR mutant (Δ) of recombinant Coi12p, and with (+) or without (−) ATP and the ATP regeneration system. The Twi1p-containing complex was immunoprecipitated with an anti-Twi1p antibody, and co-precipitated 27-nt RNAs were detected by autoradiography. The intensities of the bands in the different conditions relative to the sample with wild-type Coi12p and without an ATP supply (1^st^ column) are shown on the top. A representative experiment is shown on the bottom.

Cell lysate from *COI12*KO cells at 3 hpm was incubated with (+) or without (−) hexokinase followed by radiolabeled double-stranded 27-nt RNAs and recombinant wild-type Coi12p. Loaded RNA was analyzed as in (B). The intensities of the bands relative to the sample without hexokinase are shown.

Cell lysate from *DCL1*KO cells at 3 hpm was incubated with radiolabeled double-stranded 27-nt RNAs and then with (+) or without (−) ATP and the ATP regeneration system, the HSP70 inhibitor PES, and the HSP90 inhibitor 17-AAG. Loaded RNA was analyzed as in (B). The intensities of the bands relative to the sample without ATP and any inhibitors (1^st^ column) are shown on the top.

Cell lysate from *COI12*KO cells at 3 hpm was incubated with radiolabeled double-stranded 27-nt RNAs, recombinant wild-type Coi12p, with (+) or without (−) ATP, and with (+) or without (−) ATP-γ-S. Loaded RNA was analyzed as in (B). The intensities of the bands relative to the sample with ATP (the 1^st^ lane) are shown on the top.

GST-tagged wild-type Coi12p (GST-Coi12p) or GST-tagged Coi12p lacking the TPR domain (GST-Coi12p-ΔTPR) was incubated with His-tagged wild-type Hsp82p (His-Hsp82p) or His-tagged Hsp82p lacking the C-terminal MEDVD sequence (His-Hsp82p-ΔC) and affinity-purified with glutathione-coupled beads. Proteins before (input) or after (GST pull-down) the purification were analyzed by Western blot using anti-GST (top) and anti-His (bottom) antibodies. The positions of GST-Coi12p, GST-Coi12p-ΔTPR, His-Hsp82p, and His-Coi12p-ΔC are marked with arrowheads.

Data information: In (A–E), the standard deviation (SD) between technical replicates is indicated.

To understand the importance of the different regions of Coi12p, we analyzed the loading-promoting activity of different Coi12p truncation mutants (Fig5A). A cell lysate was prepared from *COI12* KO cells and incubated with the radiolabeled 27-nt RNA duplex and wild-type or truncated mutants of recombinant Coi12p. Then, Twi1p was immunoprecipitated with an anti-Twi1p antibody, and the co-precipitated RNA was analyzed (Fig5A). No detectable 27-nt RNA was co-precipitated with Twi1p from the *COI12* KO cell lysate (Fig5A, w/o), and the addition of recombinant Coi12p restored the co-precipitation of small RNAs with Twi1p (Fig5A, WT), indicating that the *COI12* KO cell lysate contains all the machinery necessary for scnRNA loading, except Coi12p.

A similar amount of the labeled RNA was co-precipitated with Twi1p when wild-type Coi12p or Coi12p-ΔTPR, in which the C-terminal region containing the entire TPR domain was deleted, were added to the *COI12* KO cell lysate (Fig5A, WT, ΔTPR). These results indicate that contrary to our expectation of a co-chaperone function for Coi12p, the TPR domain of Coi12p is not required for the loading of scnRNAs into Twi1p under the conditions of our cell lysate-based *in vitro* assay.

The Coi12p fragment corresponding to amino acids 129–381, which contains two FKBDs, was sufficient to promote small RNA loading into Twi1p (Fig5A, N2). The fragment containing amino acids 1–254 did not support loading (Fig5A, N1), and the fragment spanning the amino acids 196–381 only weakly promoted loading in the *COI12* KO cell lysate (Fig5A, N3). These results indicate that both FKBDs are important for the loading-promoting activity of Coi12p.

### The TPR domain of Coi12p is required for ATP-dependent enhancement of scnRNA loading in cell lysates

The finding that the deletion of the TPR domain of Coi12p did not show any obvious impact on scnRNA loading in cell lysates (Fig5A) was surprising in light of the conserved molecular architecture of Coi12p as a co-chaperone and the role of the TPR domain as an essential interaction module between co-chaperones and Hsp70/90 chaperones in many eukaryotes. In the loading assay above, the cell lysate was not supplied with ATP. The concentration of ATP in the *DCL1* and *COI12* KO cell lysates, as measured by a luciferase-coupled ATP assay system, was approximately 0.1–1 μM. This value is much lower than the cellular concentration of ATP in different organisms, which ranges from approximately 0.5 to 10 mM (Albe *et al*, 1990). The low ATP concentration in the cell lysates was most likely due to the quick turnover of endogenous ATP during the preparation of the lysates. Therefore, we may have failed to detect a role of the TPR domain of Coi12p in scnRNA loading because little ATP was available for the action of Hsp70 and/or Hsp90 in the loading assay above.

**Figure 6:**
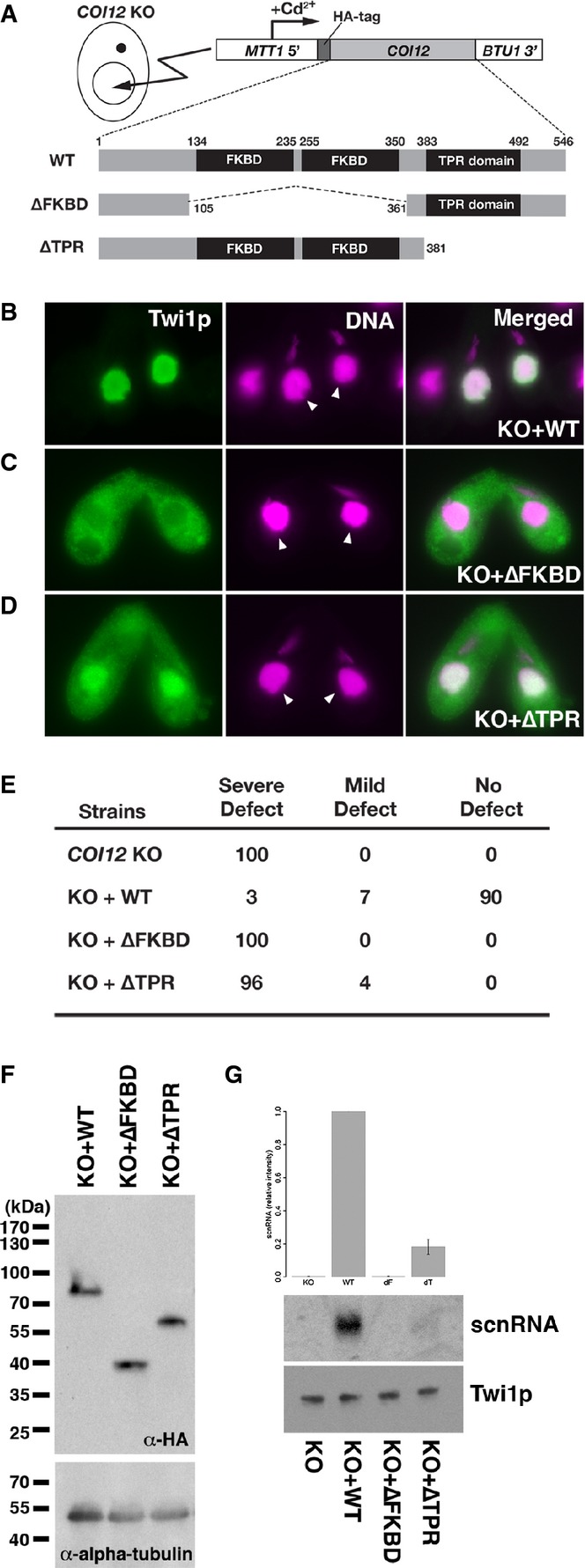
Both FKBDs and the TPR domain of Coi12p are important for scnRNA loading *in vivo*

To test this possibility, a cell lysate from *COI12* KO cells was prepared, and recombinant Coi12p (wild-type or ΔTPR) and radiolabeled 27-nt dsRNA were added and incubated in the presence or absence of ATP (final concentration of 0.4 mM) and an ATP regeneration system that includes creatine kinase and creatine monophosphate. Because ATP chelates Mg^2+^, we substituted ATP and the ATP regeneration system with 0.4 mM EDTA for ATP-negative conditions. As shown in Fig5B, supplementation with ATP further enhanced the wild-type Coi12p-mediated loading of small RNAs into Twi1p (compare “W, −” and “W, +” lanes in Fig5B). In contrast, this enhanced effect by ATP was not detected when Coi12p-ΔTPR was added to the cell lysate (compare “ΔT, −” and “ΔT, +” lanes in Fig6A). These results indicate that the TPR domain is important for the scnRNA loading-enhancing activity of Coi12p in the presence of ATP.

A schematic drawing of the *in vivo* functional analysis of *COI12* mutants. The expression constructs from which HA-tagged Coi12p proteins (wild-type [WT] or the mutant proteins lacking the two FKBDs [ΔFKBD] or the TPR domain [ΔTPR]) are expressed under the control of the cadmium-inducible *MTT1* promoter were introduced into the MACs of *COI12*KO cells.

Cells containing the expression constructs shown in (A) were mated with *COI12*KO cells in the presence of cadmium ions. The localization of Twi1p was analyzed by immunofluorescence staining using an anti-Twi1p antibody at 3 hpm (B–D). In (B–D), the parental MACs are marked with arrowheads. DNA elimination was analyzed by DNA FISH with probes against Tlr1 IESs at 36 hpm (E). Defects in DNA elimination were categorized as described for Fig1C. The expression of the different Coi12p mutants was analyzed by Western blot using an anti-HA antibody (F, top). Alpha-tubulin was analyzed as a loading control (F, bottom). The Twi1p-containing complex was immunoprecipitated with an anti-Twi1p antibody. Precipitated scnRNA was separated on a denaturing gel and visualized by the nucleic acid staining dye GelRed (G, top and middle). The intensities of bands relative to the sample with the mating expressing wild-type Coi12p (KO+WT) are shown on the top, and a result of a representative experiment is shown in the middle. Precipitated Twi1p was analyzed by Western blot using an anti-Twi1p antibody (bottom). In (G), the standard deviation (SD) between technical replicates is indicated.

The finding that ATP boosts Coi12p-mediated scnRNA loading led us to suspect that the observed scnRNA loading activity in cell lysates without ATP supplementation (Figs4 and 5A) could be dependent on some residual ATP. To test this possibility, we pre-treated a *COI12* KO cell lysate with hexokinase to deplete ATP. Although this hexokinase treatment reduced the ATP concentration in the cell lysate to below the detection limit of our assay (< 10 pM; data not shown), it did not significantly inhibit the scnRNA loading activity of wild-type Coi12p in the *COI12* KO cell lysate (Fig5C). Therefore, the residual amount of ATP plays little role in the scnRNA loading activity of Coi12p without an ATP supply. We conclude that Coi12p has two different modes of scnRNA loading-promoting activity: an ATP-independent activity that does not rely on the TPR domain and an ATP-dependent activity that requires the TPR domain.

### Hsp90 is necessary for the ATP-dependent loading-promoting activity

The ATP-dependent loading-promoting activity of Coi12p is reminiscent of the co-chaperone-dependent loading of small RNAs into Argonaute proteins in other eukaryotes (Iki *et al*, 2010, 2012; Olivieri *et al*, 2012; Preall *et al*, 2012; Xiol *et al*, 2012; Martinez *et al*, 2013). Therefore, we hypothesized that Coi12p acts as a co-chaperone of Hsp70 and/or Hsp90 to enhance the loading of scnRNAs to Twi1p.

To assess the importance of Hsp70 and Hsp90 in the scnRNA loading process, we tested the effects of Hsp70 and Hsp90 inhibitors on scnRNA loading. Cell lysates from *DCL1* KO cells, which contain endogenous Coi12p, were mixed with or without the Hsp70 inhibitor 2-phenylethynesulfonamide (PES) or the Hsp90 inhibitor 17-allylamino-17-demethoxygeldanamycin (17-AAG), incubated with the radiolabeled 27-nt RNA duplex, and incubated with or without ATP and the ATP regeneration system. As shown in Fig5D, the amount of 27-nt RNA that co-precipitated with Twi1p did not change after the addition of PES (compare lanes 1 and 2) or 17-AAG (compare lanes 1 and 3) in the absence of an ATP supply in the *DCL1* KO cell lysate. As described above, the presence of ATP enhanced the loading of small RNAs into Twi1p (compare lanes 1 and 4 in Fig5D). 17-AAG (lane 6), but not PES (lane 5), abolished this enhancement, indicating that Hsp90, but not Hsp70, is necessary for the ATP-dependent scnRNA loading-promoting activity in *Tetrahymena* cell lysates.

ATP hydrolysis by Hsp90 facilitates the loading of siRNAs into Argonaute proteins in mammals and flies. However, ATP hydrolysis is not necessary for the loading of siRNAs into tobacco AGO1. Therefore, we next asked whether ATP hydrolysis is necessary for the loading of scnRNAs into Twi1p. A cell lysate from *COI12* KO cells was prepared, and recombinant wild-type Coi12p and the radiolabeled 27-nt RNA duplex were added and incubated with ATP or the non-hydrolyzable ATP analog ATP-gamma-S. Compared to ATP, ATP-gamma-S did not inhibit, but rather slightly enhanced, the loading of small RNAs into Twi1p (Fig5E). This result indicates that as in plants, the binding of ATP to Hsp90, but not its hydrolysis, is important for enhancing loading of scnRNAs into Twi1p in *Tetrahymena*.

### Coi12p interacts with Hsp90

The requirements of Hsp90 and the TPR domain of Coi12p for the ATP-dependent scnRNA loading-promoting activity (Fig5B and D) suggest that Coi12p acts as an Hsp90 co-chaperone during this process. To further address this possibility, we analyzed the direct interaction between Coi12p and Hsp82p, the *Tetrahymena* homolog of Hsp90 (Williams & Nelsen, 1997), by GST pull-down assays using recombinantly expressed GST-tagged Coi12p (GST-Coi12p) and His-tagged Hsp82 (His-Hsp82p). His-Hsp82p was co-purified with GST-Coi12p (Fig6E, lane 4), indicating that Coi12p can indeed directly interact with Hsp82p.

The TPR domains of Hsp90 co-chaperones interact with the C-terminal MEEVD motif of Hsp90 proteins in other eukaryotes (Scheufler *et al*, 2000). *Tetrahymena* Hsp82p has a similar C-terminal motif (MEDVD; Supplementary Fig S4C). To assess the importance of the TPR domain of Coi12p and the C-terminal motif of Hsp82p in the Coi12p–Hsp82p interaction, GST pull-down assays were performed using either GST-Coi12p lacking the TPR domain (GST-Coi12p-ΔTPR) and His-Hsp82p or GST-Coi12p and His-Hsp82p lacking the C-terminal MEDVD sequence (His-Hsp82p-ΔC). Hsp82p was not co-purified with Coi12p in either assay (Fig5F, lanes 5, 6), indicating that the Coi12p–Hsp82p interaction is mediated by the TPR domain of Coi12p and the C-terminal motif of Hsp82p. Together, these results support the idea that Coi12p is an Hsp90 co-chaperone.

### Coi12p lacking the TPR domain partially restores the scnRNA loading defect of *COI12* KO cells *in vivo*

To understand the role of different regions of Coi12p *in vivo*, we used a genetic rescue system in which the non-essential *BTU1* locus (Gaertig *et al*, 1994) of the parental MAC in the *COI12* KO strains was replaced with a cassette expressing an HA-tagged *COI12* gene under the control of the cadmium-inducible *MTT1* promoter (Fig6A; Shang *et al*, 2002). Expression of the wild-type Coi12p (Fig6A, WT) in the *COI12* KO strain by this rescue system restored the parental MAC localization of Twi1p (compare Fig6B with Fig3B) and DNA elimination (Fig6E, KO+WT), indicating that the wild-type Coi12p expressed from the gene expression cassette is sufficient to restore all essential steps of DNA elimination in *COI12* KO cells. Therefore, the rescue system using the *COI12* KO strains and the gene expression cassette can be used to assay the *in vivo* activities of Coi12p mutants.

Next, we introduced gene expression cassettes for Coi12p-ΔFKBD, the Coi12p mutant lacking two FKBDs (Fig6A, ΔFKBD), and for Coi12p-ΔTPR, which lacked the TPR domain (Fig6A, ΔTPR). The expression of Coi12p-ΔFKBD did not restore the parental MAC localization of Twi1p (Fig6C) and DNA elimination (Fig6E, KO+ΔFKBD). In contrast, the expression of Coi12p-ΔTPR (Fig6A, ΔTPR) partially restored the parental MAC localization of Twi1p (Fig6D) and very weakly supported DNA elimination (Fig6E, KO+ΔTPR). These defects were not caused by reduced protein stabilities of the Coi12p mutants because a similar amount of Coi12p was detected in the *COI12* KO strains expressing wild-type Coi12p and the Coi12p mutants (Fig6F).

scnRNAs were not co-precipitated with Twi1p in the cells expressing Coi12p-ΔFKBD (Fig6G, ΔFKBD). In contrast, a small amount of scnRNAs was co-purified with Twi1p in the cells expressing Coi12p-ΔTPR (Fig6G, ΔTPR). Therefore, the FKBDs of Coi12p are essential for the loading of scnRNAs into Twi1p *in vivo*, whereas Coi12p lacking the TPR domain still retains some activity to support the loading of scnRNAs into Twi1p *in vivo*. These results suggest that not only in cell lysates but also *in vivo*, Coi12p has two modes of scnRNA loading activities: One is dependent on the TPR domain, and the other is independent of the TPR domain. Together with the fact that Coi12p-ΔTPR does not bind to Hsp90 (Fig5F) and only supports Hsp90- and ATP-independent scnRNA loading *in vitro* (Fig5B), these results suggest that scnRNAs can be loaded into Twi1p in an Hsp90- and ATP-independent manner *in vivo* in *Tetrahymena*.

### Coi12p counteracts the negative loading regulator Giw1p

**Figure 7:**
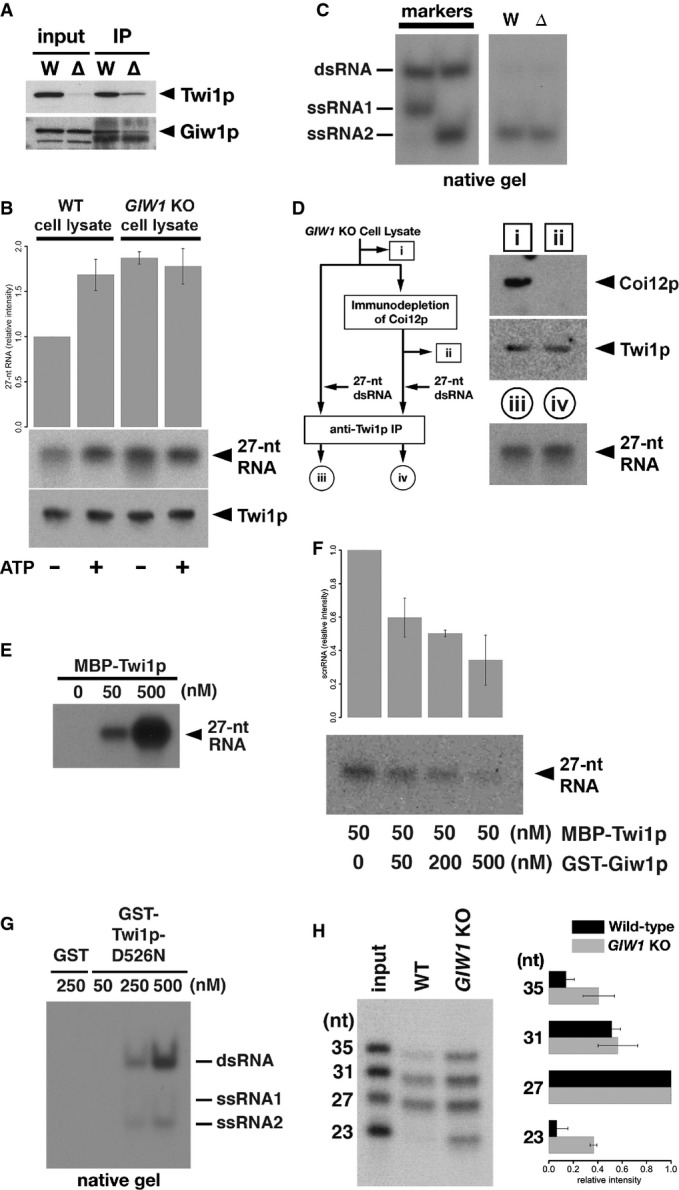
Coi12p counteracts the negative loading regulator Giw1p

We previously showed that Giw1p directly interacts with Twi1p bound by single-stranded but not by double-stranded scnRNA and that this interaction is necessary for the MAC localization of Twi1p (Noto *et al*, 2010). In the same report, we showed that Giw1p stayed bound to Twi1p even after complete degradation of scnRNAs by RNase A treatment of wild-type cell lysate, although the biological importance of this interaction was not clear. We confirmed this observation by testing co-precipitation of Twi1p and Giw1p in *DCL1* KO cells, in which Twi1p is in an unloaded state due to lack of scnRNAs (Malone *et al*, 2005; Mochizuki & Gorovsky, 2005). The Twi1p-containing complex was immunoprecipitated from wild-type and *DCL1* KO cells at 3 hpm using an anti-Twi1p antibody. Lesser Twi1p was precipitated from *DCL1* KO cell than from wild-type cells (Fig7A, Twi1p, IP) due to the instability of unloaded Twi1p (Fig3E). Gwi1p was clearly co-precipitated with Twi1p in the absence of *DCL1* and the amount of precipitated Gwi1p correlated with the amount of precipitated Twi1p in wild-type and *DCL1* KO cells (Fig7A, Gwi1p, IP), indicating that Giw1p can interact with unloaded Twi1p *in vivo*. Giw1p binds to both the PAZ and Piwi domains of Twi1p (Noto *et al*, 2010). Structural analyses of Argonaute proteins indicate that their siRNA-binding cleft lies between the PAZ and Piwi domains (Wang *et al*, 2008; Nakanishi *et al*, 2012; Elkayam *et al*, 2012). Therefore, we suspected that Giw1p inhibits scnRNA loading by binding to unloaded Twi1p, and Coi12p counteracts this inhibitory activity to enhance loading.

The Twi1p-containing complex was immunoprecipitated using an anti-Twi1p antibody from wild-type (W) and *DCL1*KO (Δ) cells, and input (left) and precipitated (right) proteins were analyzed by Western blot using an anti-Twi1p antibody (top) and an anti-Giw1p antibody (bottom).

Cell lysates from wild-type (WT) or *GIW1*KO cells at 3 hpm were incubated with radiolabeled double-stranded 27-nt RNAs and with (+) or without (−) ATP and the ATP regeneration system. The Twi1p-containing complex was immunoprecipitated with an anti-Twi1p antibody, co-precipitated RNAs were separated on a denaturing gel and detected by autoradiography. The intensities of the bands in the different conditions relative to the sample with wild-type cell lysate and without ATP (1^st^ column) are shown on the top. The standard deviation (SD) between technical replicates is indicated. A result of a representative experiment is shown on the bottom.

RNAs were prepared from cell lysates from wild-type (W) or *GIW1*KO (Δ) cells with ATP and the ATP regeneration system as in (A) but were separated in a native gel. The positions of double-stranded 27-nt RNAs (dsRNA) and each strand of RNA used to form the 27-nt RNA duplex (ssRNA1 and ssRNA2) are indicated at the left.

Coi12p was immunodepleted from *GIW1*KO cell lysate at 3 hpm, and the lysate was incubated with radiolabeled double-stranded 27-nt RNAs. As a control, *GIW1*KO cell lysate without Coi12p depletion was used for the loading assay. The protein samples (i, ii) correspond to the lanes shown in the top right panel. Coi12p and Twi1p in the lysates were detected by Western blot. The Twi1p-containing complex was immunoprecipitated with an anti-Twi1p antibody, and co-precipitated 27-nt RNAs were detected by autoradiography. The RNA samples (iii, iv) correspond to the lanes shown in the bottom right panel.

The indicated concentration of MBP-tagged Twi1p (MBP-Twi1p), which was recombinantly expressed in *E. coli*, was incubated with the radiolabeled double-stranded 27-nt RNAs. Loaded RNA was analyzed by immunoprecipitating MBP-Twi1p with an anti-Twi1p antibody and separated on a denaturing gel, followed by autoradiographic detection.

MBP-Twi1p (50 nM) was incubated with the indicated concentrations of the recombinantly expressed GST-Giw1p and with radiolabeled 27-nt RNA duplexes. Loaded RNA was analyzed as in (C). The intensities of the bands in the different conditions relative to the sample without GST-Giw1p (1^st^ column) are shown on the top. The standard deviation (SD) between technical replicates is indicated. A result of a representative experiment is shown on the bottom.

The indicated concentration of recombinantly expressed GST-tagged Twi1p in which aspartic acid 526 was replaced by asparagine (GST-Twi1p-D526N) or GST alone was incubated with radiolabeled double-stranded 27-nt RNAs. Loaded RNA was analyzed by immunoprecipitating GST-Twi1p-D526N with an anti-Twi1p antibody and separating on a native gel, followed by autoradiographic detection. The positions of double-stranded 27-nt RNAs (dsRNA) and each strand of RNA used to form the 27-nt RNA duplex (ssRNA1 and ssRNA2) are indicated at the right.

Cell lysates from wild-type (WT) or *GIW1*KO cells at 3 hpm were incubated with a mixture of radiolabeled double-stranded 23-, 27-, 31-, and 35-nt RNAs, ATP, and the ATP regeneration system. The Twi1p-containing complex was immunoprecipitated with an anti-Twi1p antibody, and co-precipitated RNAs were detected by autoradiography. The intensities of each RNA band relative to the 27-nt RNA were normalized to the input sample and are shown on the right. The standard deviation (SD) between technical replicates is indicated. A representative experiment is shown on the left.

First, to test whether Giw1p inhibits the loading of scnRNA duplexes into Twi1p, we performed scnRNA loading assays using cell lysates from wild-type and *GIW1* KO cells. As shown in Fig7A, more scnRNAs were co-precipitated with Twi1p in a *GIW1* KO cell lysate than in the wild-type cell lysate without an ATP supply (compare lanes 1 and 3 of Fig7A), suggesting that Giw1p indeed has an inhibitory effect on scnRNA loading into Twi1p in cell lysates. Although the addition of ATP enhanced loading in the wild-type cell lysate (compare lanes 1 and 2 of Fig7A), the presence of ATP did not further enhance the loading of scnRNAs in the *GIW1* KO cell lysate (compare lanes 3 and 4 of Fig7A). These results indicate that the primary role of the ATP-dependent scnRNA loading-enhancing activity in the cell lysate is to counteract the loading inhibitory effect of Giw1p.

Next, to understand the relationship between the ATP-independent loading-promoting activity of Coi12p and Giw1p, scnRNA loading assays were performed with the *GIW1* KO cell lysate without ATP supplementation and with or without immunodepletion of Coi12p (Fig7B). We found that the removal of Coi12p from the *GIW1* KO cell lysate did not inhibit the loading of 27-nt RNA duplexes into Twi1p (Fig7B). Therefore, we conclude that the primary role of Coi12p in scnRNA loading, regardless of the presence or absence of ATP, is to counteract the loading inhibitory activity of Giw1p.

### scnRNAs are loaded into Twi1p without co-factors *in vitro*

Because scnRNA duplexes could be loaded into Twi1p without Coi12p in the *GIW1* KO cell lysate, we suspected that no co-factor is necessary for scnRNA loading in the absence of Giw1p. To test this possibility, we performed a simple two-component loading assay with recombinantly expressed MBP-tagged Twi1p (MBP-Twi1p) and the radiolabeled 27-nt RNA duplex. These components were mixed, MBP-Twi1p was immunoprecipitated with an anti-Twi1p antibody, and the co-precipitated small RNA was analyzed. As shown in Fig7C, the labeled small RNAs were indeed co-precipitated with MBP-Twi1p, indicating that scnRNAs can be loaded into Twi1p without any co-factor.

Next, to ask whether we can reconstitute the loading inhibition exerted by Giw1p in this *in vitro* assay system, we first incubated MBP-Twi1p with recombinantly expressed GST-Giw1p and then performed a similar loading assay with the radiolabeled 27-nt RNA duplex. As shown in Fig7D, the loading of the 27-nt RNA into Twi1p was strongly inhibited by the addition of Giw1p (Fig7E). This was not simply due to degradation of the RNA duplex by an RNase contaminant in the recombinant GST-Giw1p because incubation of the 27-nt RNA duplex with the highest concentration of GST-Giw1p used for the assay did not greatly reduce the amount of the 27-nt RNA (Supplementary Fig S6). These results indicate that Giw1p alone is sufficient to inhibit the loading of scnRNAs into Twi1p *in vitro*.

Single-stranded siRNAs can be loaded into fly and mammalian Argonaute proteins without any co-factors (Martinez *et al*, 2002; Rivas *et al*, 2005; Miyoshi *et al*, 2005), and we previously showed recombinant Twi1p can be loaded with single-stranded scnRNAs (Noto *et al*, 2010). Therefore, we suspected that a small amount of single-stranded small RNA dissociated from the 27-nt RNA duplex during our loading assay and was subsequently loaded into recombinant Twi1p. To test the direct loading of small RNA duplexes into Twi1p, we used the Twi1p-D526N mutant, which lacks the endonuclease (Slicer) activity and thus cannot efficiently dissociate one of the two strands of scnRNAs *in vivo* (Noto *et al*, 2010). If an RNA duplex is loaded into Twi1p, we expect that it will remain double-stranded in Twi1p-D526N. We mixed GST-tagged Twi1p-D526N (GST-Twi1p-D526N) and the radiolabeled 27-nt RNA duplex, GST-Twi1p-D526N was immunoprecipitated with an anti-Twi1p antibody, and the co-precipitated small RNAs were analyzed in a native, instead of denaturing, gel. We found that the RNAs that co-precipitated with GST-Twi1p-D526N were mostly double-stranded (Fig7E), strongly indicating that small RNA duplexes can be directly loaded into Twi1p without any co-factors. All of these results further support our conclusion above that the primary role of Coi12p in loading is to counteract the loading inhibitory activity of Giw1p.

### Giw1p is necessary for the sorting of scnRNAs to Twi1p

We hypothesized that Giw1p might be necessary for the selective loading (sorting) of scnRNAs into Twi1p. However, as we previously showed, the Twi1p-bound small RNA populations in wild-type cells and *GIW1* KO cells are indistinguishable by denaturing gel electrophoresis (Noto *et al*, 2010; see also Supplementary Fig S7A). We confirmed this result by sequencing those small RNAs (Supplementary Fig S7B). It is possible that we could not detect any defect in sorting in *GIW1* KO cells *in vivo* because the majority of small RNAs are scnRNAs at the time of Twi1p–scnRNA loading (Mochizuki *et al*, 2002; Noto *et al*, 2010).

Therefore, we tested the importance of Giw1p in sorting by an *in vitro* experiment. We mixed radiolabeled 23-, 27-, 31-, and 35-nt RNA duplexes and compared their loading into Twi1p in cell lysates prepared from wild-type and *GIW1* KO cells. Note that the 27- and 31-nt RNAs correspond to scnRNAs (26- to 32-nt; Mochizuki *et al*, 2002; Mochizuki & Kurth, 2013), whereas the 23-nt RNA corresponds to the constitutively expressed siRNAs (23- to 24-nt; Lee & Collins, 2006). In the wild-type cell lysate, 27- and 31-nt RNAs were mainly co-precipitated with Twi1p, whereas 23- and 35-nt RNAs were largely excluded from the Twi1p-bound RNA fraction (Fig7F, WT). This result indicates that a sorting mechanism preferentially loads scnRNA-sized RNA duplexes into Twi1p in the wild-type cell lysate. In contrast, all of the RNAs were co-precipitated with Twi1p in the *GIW1* KO cell lysate (Fig7F, *GIW1* KO), although, possibly due to some structural restriction of Twi1p, the 27- and 31-nt RNAs were still preferentially associated with Twi1p. Therefore, we conclude that the Giw1p–Coi12p antagonism plays an important role in sorting scnRNA duplexes to Twi1p.

## Discussion

In this study, we found that the *Tetrahymena* Hsp90 co-chaperone Coi12p is necessary for the loading of scnRNAs into the Argonaute protein Twi1p (Fig3G) and thus for DNA elimination *in vivo* (Fig1C). Coi12p has two modes of scnRNA loading-promoting activities *in vitro*: ATP-dependent activity, which requires Hsp90 and the TPR domain of Coi12p, and ATP-independent activity, in which Hsp90 and the TPR domain are dispensable (Fig5). Both activities facilitate scnRNA loading by counteracting the Twi1p-binding protein Giw1p, which is important for sorting scnRNAs to Twi1p (Fig7). Furthermore, the TPR domain-dependent and TPR domain-independent scnRNA loading activities also function *in vivo* (Fig6).

### ATP-independent loading of siRNA duplexes into an Argonaute protein

Although the Coi12p mutant lacking the TPR domain (Coi12p-ΔTPR) does not interact with Hsp90 (Fig5F) and can only mediate ATP- and Hsp90-independent loading of scnRNA duplexes to Twi1p in cell lysates (Fig5B), Coi12p-ΔTPR can still support scnRNA loading and DNA elimination *in vivo*, albeit less efficiently than the wild-type Coi12p (Fig6). These results indicate that in *Tetrahymena*, ATP- and Hsp90-independent loading of siRNA duplexes can occur *in vivo* and that this loading process is not simply a bypass mechanism but plays a physiological role in this organism. Similar to our observations, mutations in the conserved residues of the TPR domain of the fly co-chaperone Shutdown compromise only the secondary biogenesis of piRNAs, although a null mutation of *shutdown* abolishes both primary and secondary piRNA biogenesis (Olivieri *et al*, 2012), indicating that Shutdown might also have an Hsp90- and ATP-independent role in piRNA biogenesis.

Although ATP and Hsp90 clearly enhance the loading of siRNAs and miRNAs into Argonaute proteins in flies (Iwasaki *et al*, 2010), mammals (Yoda *et al*, 2010), and plants (Iki *et al*, 2010), whether an Hsp90- and ATP-independent RISC assembly pathway exists *in vivo* in any eukaryote has not been shown. Therefore, it would be important to find a mutant that, similar to Coi12p-ΔTPR, allows the genetic separation of the ATP-dependent and ATP-independent loading processes of double-stranded small RNAs to determine whether the latter occurs in other eukaryotes *in vivo*.

In addition to the ATP-independent loading-enhancing activity, an Hsp90- and ATP-dependent activity promotes loading of scnRNAs into Twi1p in *Tetrahymena,* as in many other eukaryotes (Fig6A–C). The functional distinction of these ATP-dependent and ATP-independent activities, if any, is not clear. Depending on the cellular energy states, two modes of activity may adjust the rate of downstream processes, such as the nuclear import of Twi1p (Noto *et al*, 2010) and homology-dependent degradation of scnRNAs, which is regulated by the putative ATP-dependent RNA helicase Ema1p (Aronica *et al*, 2008; Schoeberl *et al*, 2012). Alternatively, the two modes may be necessary to maintain the environmental robustness of the loading process, such that the ATP-dependent activity might be essential for loading in some environments in which the dynamics of molecules are sub-optimal for scnRNA loading.

Because double-stranded scnRNAs loaded into Twi1p were mostly converted into single-stranded RNAs in the cell lysate even without ATP addition (Fig4D), ATP is dispensable not only for loading but also for passenger strand removal. However, in our loading assay, the loaded scnRNAs were detected by immuno-precipitating Twi1p, and thus, we most likely observed the only end point of the loading process. Therefore, it is possible that the presence of ATP accelerates passenger strand removal, but our assay is not suitable for dissecting the role of ATP in this process.

### An Argonaute-binding protein regulates the siRNA loading process

In the absence of the Twi1p-binding protein Giw1p, a scnRNA duplex can be loaded into Twi1p without any co-factors (Fig7C–E), indicating that Twi1p is intrinsically able to be loaded with small RNA duplexes but that Giw1p, which interacts with the PAZ and Piwi domains of Twi1p (Noto *et al*, 2010), may act as a “lid” that inhibits the loading of scnRNA duplexes into Twi1p. This idea seems to contrast the previously proposed mechanism of the loading of miRNAs and siRNAs into Argonaute proteins in mammals, flies, and plants, in which ATP-bound Hsp90 is suggested to promote loading by inducing a conformational change of the Argonaute proteins to accommodate a bulky small RNA duplex (Kawamata & Tomari, 2010). However, these two mechanisms may not be mutually exclusive because the removal of Giw1p may be accompanied by a conformational change of Twi1p in *Tetrahymena* and/or a Giw1p-like lid might have been overlooked in previous RISC assembly assays in other eukaryotes, which were often performed with crude cell lysates. Although we could not identify any Giw1p homolog in non-*Tetrahymena* species, a functionally similar “lid” could also exist in other eukaryotes, and Hsp90 chaperone activity might be used to displace the lid to promote loading. In this context, it would be interesting to revisit Argonaute-binding proteins in metazoans to determine whether some of them are functionally equivalent to Giw1p.

We previously showed that Giw1p binds to Twi1p complexed with single-stranded, but not double-stranded, scnRNAs and that this interaction selectively promotes the MAC localization of the mature Twi1p–scnRNA complex (Noto *et al*, 2010). In this study, we showed an additional role of Giw1p: This protein interacts with unloaded Twi1p and inhibits loading of small RNAs into Twi1p. It remains unclear how unloaded Twi1p escapes from the MAC import. In addition to its interaction with Giw1p, a conformational change or a post-translational modification of Twi1p that is caused by the loading of scnRNA might be necessary for the MAC import of Twi1p.

### A Giw1p–Coi12p counteracting system for the sorting of scnRNAs

Giw1p plays an important role in the sorting of scnRNAs to Twi1p (Fig7F). Giw1p most likely protects Twi1p from incorrect loading and may be counteracted by Coi12p only for the loading of proper cargo (scnRNA duplex). Currently, how scnRNA duplexes are specifically recognized and how this recognition is coupled to loading remains unclear. In flies, the RISC loading complex (RLC), which contains Dicer and R2D2, is crucial for the loading of siRNAs into Ago2 (Liu *et al*, 2003; Pham *et al*, 2004; Liu *et al*, 2006). However, *Tetrahymena* unlikely uses a similar RLC for scnRNA loading (and thus for sorting) into Twi1p because no obvious R2D2 homolog is found in *Tetrahymena* and because Dcl1p is not necessary for the loading of exogenously supplied scnRNA duplexes into Twi1p in cell lysates (Fig4). The Dicer-independent loading of scnRNAs into Twi1p is not surprising because the loading of siRNAs into fly Ago1 and mammalian Ago proteins does not require Dicer (Martinez *et al*, 2002; Kanellopoulou *et al*, 2005; Murchison *et al*, 2005; Kawamata *et al*, 2009; Betancur & Tomari, 2012). A cytoplasmic protein that specifically binds to scnRNA duplexes could be involved in the sorting of scnRNAs to Twi1p in *Tetrahymena*. Coi12p is unlikely to be such a scnRNA duplex-binding protein because our attempts to detect a direct interaction between recombinant Coi12p and scnRNA duplexes *in vitro* have failed (data not shown), although the recombinant Coi12p expressed in *E. coli* might lack some post-translational modification critical for its binding to scnRNA duplexes. Future studies should reveal the protein that interacts with scnRNA duplexes prior to loading and how such a protein links scnRNA duplexes to the loading process.

## Materials and Methods

### Strains and culture conditions

Wild-type B2086 and CU428 strains of *Tetrahymena thermophila* were provided by Dr. P. J. Bruns (Cornell University). *DCL1* and *GIW1* knockout (KO) strains were described previously (Malone *et al*, 2005; Noto *et al*, 2010). Cells were grown in SPP medium (Gorovsky *et al*, 1975) containing 2% proteose peptone, at 30°C. For conjugation, growing cells (∽5–7 × 10^5^/ml) of two different mating types were washed, pre-starved (∽12–24 h), and mixed in 10 mM Tris (pH 7.5) at 30°C.

### Gene expression and scnRNA analyses

Total RNA was extracted from wild-type (B2086 and CU428) strains and analyzed by RT–PCR as described previously (Aronica *et al*, 2008) using the primers listed in Supplementary Table S1. RNA expression data from microarray analyses (Miao *et al*, 2009) were obtained from the *Tetrahymena* Functional Genomics Database (http://tfgd.ihb.ac.cn/). Total RNA extracted from 1.25 × 10^5^ starved and mating cells was used for Northern hybridization. Total and Twi1p-bound scnRNAs were analyzed as described previously (Noto *et al*, 2010). High-throughput sequencing of small RNAs was performed as described previously (Schoeberl *et al*, 2012; Noto *et al*, 2014), except that 16- and 32-nt RNAs were used as size markers for the gel extraction steps. The raw sequencing data and processed data sets have been deposited at the NCBI Gene Expression Omnibus (www.ncbi.nlm.nih.gov/geo/) as GSE61609.

### Production of gene KO strains and DNA elimination assays

Somatic KO strains and germline KO strains were produced as described previously (Mochizuki & Gorovsky, 2005; Aronica *et al*, 2008). Schematic drawings of the targeting constructs can be found in Supplementary Fig S2. The DNA oligos used for PCR to produce the targeting constructs are listed in Supplementary Table S2. DNA elimination of the *Tlr1* and *REP2* elements was analyzed by DNA FISH as described previously (Noto *et al*, 2010).

### Production of recombinant proteins

*COI12* and *HSP82* coding regions that were optimized for *E. coli* expression, named *COI12Ec* and *HSP82Ec*, respectively, were synthesized (GenScript). The *COI12Ec* and *HSP82Ec* sequences can be found in Supplementary File S1. DNAs corresponding to the truncated mutants of Coi12p (ΔTPR: 1–381aa; N1: 1–254aa; N2: 129–381aa, N3 196–381aa) and HSP82 lacking the C-terminal MEDVD sequence (*HSP82-ΔC*Ec) were produced by PCR using *COI12Ec* or *HSP82Ec*. The wild-type or truncated mutants of *COI12Ec* were cloned into pGEX-4T-1 or its variant, which contained a TEV cleavage site between the N-terminal GST-tag and the protein-coding sequence (gift from Dr. Tim Clausen, IMP, Austria). HSP82Ec and *HSP82-ΔC*Ec were cloned into pET28a. The production of GST-Twi1p-D526N and GST alone were described previously (Noto *et al*, 2010). To produce MBP-TEV-Twi1p or GST-Giw1p, DNA fragments encoding codon-optimized full-length *TWI1* and *GIW1* (Noto *et al*, 2010) were cloned into pMAL-c2X-TEV and pGEX4T-1 (GE Healthcare), respectively. pMAL-c2X-TEV was produced from pMAL-c2X (New England Biolabs) by adding a TEV protease cleavage sequence immediately before the EcoRI site. All recombinant proteins were expressed in *E. coli* strain BL21 (DE3). Bacteria cultures were grown at 37°C in LB with ampicillin to an OD_600_ of approximately 0.7. The expression of His-Hsp82p proteins was induced with 0.3 mM IPTG at 25°C for 3 h. For the remaining proteins, the cultures were cooled on ice for 5 min, and protein expression was induced with 0.5 mM IPTG at 18°C overnight. The harvested cells were lysed either in 20 mM Tris, 500 mM NaCl (pH 7.5), 1× complete protease inhibitor cocktail (Roche), and with or without 1 mg/ml lysozyme (for recombinant Coi12p proteins); 80 mM Tris pH 8.0, 500 mM NaCl, 0.5% Triton X-100, 0.2 mM PMSF, and 1× complete EDTA-free (Roche; for recombinant Twi1p proteins); or NiNTA buffer A (for His-tagged proteins, 50 mM NaH_2_PO_4_, 500 mM NaCl, 5 mM DTT, adjusted to pH 8). The cell lysate was incubated with glutathione–Sepharose 4B (GE Healthcare) for GST and GST-fusion proteins or with amylose resin (New England Biolabs) for MBP-fusion proteins at 4°C for 1–2 h. The beads were washed with either 20 mM Tris, 500 mM NaCl pH 7.5 (for recombinant Coi12p proteins) or 80 mM Tris pH 8.0, 500 mM NaCl (for recombinant Twi1p proteins). GST and GST-fusion proteins were eluted in 50–160 mM glutathione in the corresponding buffers. MBP-fusion proteins were eluted in 10 mM maltose, 80 mM Tris pH 8.0, and 500 mM NaCl. The eluted recombinant proteins were dialyzed against PBS and stored at −80°C. For TEV cleavage, the beads were washed twice each with 20 mM Tris, 500 mM NaCl pH 7.5, and TEV cleavage buffer (50 mM Tris, 50 mM NaCl, 1 mM EDTA, 5 mM DTT, pH 7.5) and incubated with approximately 0.1 mg/ml TEV protease in TEV cleavage buffer overnight at 4°C. His-tagged proteins were loaded onto His-Trap columns (GE Healthcare); washed with 4%, 8% and then 14% NiNTA buffer B (NiNTA buffer A including 250 mM imidazole); and finally eluted with 60% NiNTA buffer B. For further purification of Coi12p and Hsp82p proteins, the sample was loaded onto a 16/60 Superdex S-200 column (Amersham Biosciences), and size exclusion chromatography was performed with 20 mM Tris and 150 mM NaCl (pH 7.5). Fractions containing the purest Coi12 proteins were pooled, concentrated to approximately 10 mg/ml, and stored at −80°C.

### Antibodies

To generate the anti-Coi12p antibody, a rabbit was immunized with GST-Coi12p expressed in *E. coli* (see above). The rabbit polyclonal antibody for Twi1p and Giw1p was described previously (Aronica *et al*, 2008; Noto *et al*, 2010). The mouse monoclonal anti-HA antibody HA.11 clone 16B12 (Covance) was commercially available. The mouse monoclonal anti-alpha-tubulin antibody 12G10 was obtained from the Developmental Studies Hybridoma Bank at the University of Iowa.

### Peptidyl-prolyl isomerization assay

Five micrograms of alpha-chymotrypsin from bovine pancreas (Sigma) and 1 μg of recombinantly expressed Coi12p, cyclophilin A (ProSpec), or buffer only were mixed in 250 μl of PPIase reaction buffer (50 mM Tris pH 8, 100 mM NaCl) on ice. Then, 1.25 μl of 10 mM succinyl-Ala-Leu-Pro-Phe-p-nitroanilide (Sigma) was added; the solution was mixed by pipetting a few times and transferred immediately to a transparent 96-well plate (Costar). The absorbance at 390 nm was measured at 2-s intervals during 3–5 min using a Synergy H1 Hybrid Multi-Mode Microplate Reader (BioTek Instruments). The log of the difference between the absorbance at steady state and the absorbance at time *t* was plotted, and the first-order rate constant, *k* (s^−1^), was calculated from the slope of the resulting straight line.

### Preparation of ^32^P-labeled scnRNA duplexes

Two complementary (with 2-nt 3′ overhangs) RNA oligos (50 pM each) were labeled by incubating with 50 μCi of gamma-^32^P-ATP, 20 U of Optikinase (GE Healthcare), and 1× Optikinase buffer in a 50-μl reaction volume at 37°C for 30–60 min. For all of the loading assays except experiment shown in Fig7F, sR-3hit-G and sR-3hit-aG were used as RNA oligos. For the experiment shown in Fig7F, RNA23S and RNA23A were used as 23-nt RNAs, RNA27S and RNA27A were used as 27-nt RNAs, RNA31S and RNA31A were used as 31-nt RNAs, and RNA35S and RNA35A were used as 35-nt RNAs. The sequences of the RNA oligos are listed in Supplementary Table S3. Unincorporated radioactivity was removed using Sephadex G-50 Quick Spin Columns (Roche). After heat denaturing at 95°C for 1 min, two oligos were annealed in 10 mM Tris (pH 7.5), 20 mM NaCl at RT for 1 h. Then, the annealed oligos were separated by 15% native PAGE; double-stranded oligos were excised from the gel and eluted with 0.4 M NaCl by first freezing in liquid nitrogen and then incubating with shaking at 10°C overnight. The gel was removed by ULTRAFREE-MC columns (0.22 μm, Millipore), and oligos were ethanol-precipitated with GlycoBlue (Life Technologies). The pellet was dissolved in approximately 30 μl of water and stored at −20°C.

### Preparation of antibody-immobilized beads

A 200 μl suspension of Affi-Prep Protein A Support (Bio-Rad) was washed twice with 500 μl of 1× loading buffer (30 mM HEPES-KOH pH 7.4, 40 mM KOAc, and 5 mM Mg(OAc)_2_) and resuspended in 500 μl of 1× loading buffer. Fifty microliters of 10% BSA was added and incubated at RT for 30 min. Then, 100 μl of an antiserum was added, and the reaction was further incubated for 2 h. The beads were washed three times with 500 μl of 1× loading buffer, resuspended with 400 μl of 1× loading buffer, and stored at 4°C. For co-imunoprecipitation analysis of Twi1p and Giw1p, anti-Twi1p-immobilized beads were prepared as described previously (Noto *et al*, 2010).

### Loading assays with cell lysates

At 3 hpm, 5 × 10^7^ conjugating cells were collected and suspended in 1 ml of 1× loading buffer + DC (30 mM HEPES-KOH pH 7.4, 40 mM KOAc, 5 mM Mg(OAc)_2_, 5 mM DTT, and 1× complete protease inhibitors [Roche]). Cells were homogenized on ice with a Dounce homogenizer for 100 strokes, and cell debris was removed by centrifugation at 20,000 *g* at 4°C for 20 min. For ATP depletion, 100 μl of cell lysate was incubated with 20 mM glucose and 0.1 U/μl hexokinase (Sigma) for 10 min at 25°C. In place of hexokinase, 1× loading buffer was used for the control samples. For Coi12p immunodepletion, 150 μl of cell lysate was incubated with 100 μl of anti-Coi12p bead suspension (see above) at 4°C for 15 min and then with another 100 μl of fresh anti-Coi12p bead suspension at RT for 15 min. Twenty microliters of lysate was removed for Western blot analyses. One hundred microliters of cell lysate was mixed with 1 μl of RiboLock RNase inhibitor (Fermentas); 5 μl of labeled dsRNAs (total ∽3 pmol); and with or without 7.5 pmol (50 nM for the experiment shown in Fig4) or 30 pmol (200 nM for the experiment shown in Figs5 and 6) recombinant wild-type or mutant Coi12p, 1 mM 17-(Allylamino)-17-demethoxygeldanamycin (17-AAG, Sigma), 1 mM 2-phenyl-ethynesulfonamide (PES, Sigma), and 1× reaction mix (3 mM HEPES-KOH pH 7.4, 4 mM KOAc, 0.5 mM Mg(OAc)_2_, 0.5 mM DTT, 0.4 mM ATP, 10 mM creatine monophosphate (Sigma), and 12 mU/μl creatine phosphokinase (Sigma)). In the ATP control samples, 0.4 mM EDTA in 1× loading buffer + DC was used instead of 1× reaction mix. The reaction volume was adjusted to 150 μl by adding 1× loading buffer + DC. DMSO was added to the control samples of the inhibitor assays to adjust the concentration of DMSO to be equal to the corresponding samples. After incubation at RT for 15 min, a 50-μl suspension of anti-Twi1p beads was added, and the samples were incubated at RT for 45 min. The beads were washed three times with 0.5 ml of 1× loading buffer for 10 min each and then resuspended with 90 μl of 1× loading buffer. Half of the bead suspension was used for RNA extraction with 1 ml of TRIzol (Life Technologies). The other half of the beads was used for protein extraction with an equal volume of 2× SDS sample buffer. The extracted RNA was dissolved in 7 μl of 50% formamide and heat-denatured. Then, 0.7 μl of 10× loading dye was added, and the RNAs were separated on a 15% sequencing gel and detected by autoradiography. The protein sample was incubated at 95°C for 5 min and used for Western blotting.

### ATP assay

The ATP concentration in cell lysates was analyzed using an ENLITEN ATP Assay System (Promega). Ten-fold serial dilutions of the cell lysate (×1 to ×1/100,000) and ATP standard (10^−11^–10^−16^ M) were prepared with 1× loading buffer + DC, and 10 μl of each was mixed with 100 μl of rL/L solution. Luminescence was measured using a Synergy H1 Hybrid Multi-Mode Microplate Reader (BioTek Instruments). A standard curve was made with the ATP standards in each experiment, and the concentration of ATP in the cell lysate was estimated from the standard curve.

### GST pull-down assay

Five micrograms of GST-tagged wild-type Coi12p (GST-Coi12p) or GST-tagged Coi12p lacking the TPR domain (corresponding to amino acids 382–546 of Coi12p; GST-Coi12p-ΔTPR) was mixed with 5 μg of His-tagged wild-type Hsp82 (His-Hsp82p) or His-tagged Hsp82 lacking the last five amino acids (His-Hsp82p-ΔC) in 22 μl of GST-PD buffer (20 mM Tris pH 7.5, 150 mM NaCl, 0.05% Triton X-100). Two microliters of the mix was mixed with 18 μl of 1× SDS–PAGE buffer and used as the “input” samples. The remainder of the samples was incubated at 4°C for 1 h. In parallel, 50 μl of a glutathione–Sepharose 4B bead suspension (GE Healthcare) was incubated with GST-PD buffer containing 0.1% BSA at 4°C for 1 h, and then, the beads were washed twice with GST-PD buffer. The protein complex containing the GST-tag was affinity-purified by incubating with the beads at 4°C for 1 h, followed by washing three times with GST-PD buffer at 4°C for 10 min each. The purified proteins were eluted from the beads by incubating with 40 μl of 1× SDS–PAGE buffer at 95°C for 5 min (“GST pull-down” sample). Ten microliters of the input samples and GST pull-down samples was analyzed by Western blotting using an anti-GST antibody (BD Bioscience) and an anti-6xHis antibody (Penta-His antibody, 5-PRIME).

### *In vivo* functional analysis of *COI12* mutants

The pBMNB1-HA vector was established for *Tetrahymena* gene expression (Supplementary File S1), which was designed to express a gene from the non-essential *BTU1* locus under the control of the cadmium-inducible *MTT1* promoter. The wild-type or truncated mutants (ΔFKBD and ΔTPR) of *COI12* coding genomic DNA were produced by PCR using genomic DNA from wild-type cells and were inserted into the BamHI and NheI sites of pBMNB1-HA. Then, the plasmid DNAs were linearized by XhoI and introduced into *COI12* KO cells. The *COI12* KO locus has the *neo4* cassette (Mochizuki, 2008), which expresses the neomycin resistance (neo-r) gene only when cadmium is supplied in the medium. In contrast, pBMNB1-HA has the *neo5* cassette (Busch *et al*, 2010), which constitutively expresses neo-r gene. Therefore, *COI12* KO cells with the introduced constructs were selected with paromomycin (which is inactivated by the product of neo-r gene) in the absence of cadmium. Phenotypic assortment was achieved by culturing cells with increasing concentrations of paromomycin.

### *In vitro* loading assay

MBP-TEV-Twi1p or GST-Twi1p-D526N was incubated with 1.5 pmol of ^32^P-labeled 27-nt RNA duplex (see above) in 100 μl of 1× loading buffer (30 mM HEPES-KOH pH 7.4, 40 mM KOAc, 5 mM Mg(OAc)_2_, 5 mM DTT) containing 10 μg of yeast RNA, 0.1 mg of BSA, and 40 U of RiboLock RNase Inhibitor (Thermo Scientific) at RT for 15 min. The reactions were then incubated with anti-Twi1p antibody-coupled Affi-Prep protein A support (Bio-Rad) at RT for 45 min. The beads were washed three times with 1× loading buffer at RT for 10 min and then incubated with TRIzol (Invitrogen) at RT for 5 min. For denaturing PAGE analyses, isolated RNA was dissolved in 6 μl of 50% formamide, and RNA was heat-denatured. For native PAGE analyses, RNA was dissolved in 6 μl of water. RNA was mixed with 1 μl of loading dye, separated by denaturing or native PAGE, and detected by autoradiography. For the loading assay including GST-Giw1p, 5 pmol of MBP-TEV-Twi1p was pre-incubated with 5, 20, or 50 pmol of GST-Giw1p in 100 μl of 1× loading buffer containing 10 μg of yeast RNA; 0.1 mg of BSA; and 40 U of RiboLock RNase Inhibitor at RT for 45 min. After the pre-incubation, the loading of the ^32^P-labeled 27-nt RNA duplex was analyzed as described above.

## Supporting Information

Supplementary File S1

Review Process File
