# Supplementary material for: A Tetrahymena Hsp90 co-chaperone promotes siRNA loading by ATP-dependent and ATP-independent mechanisms
Source: EMBO J. 2015 Jan 14;34(4):559–77. doi: 10.15252/embj.201490062 (PMC4331008; doi:10.15252/embj.201490062)
Supplement: Supplementary file 1 [file embj0034-0559-sd1.pdf]

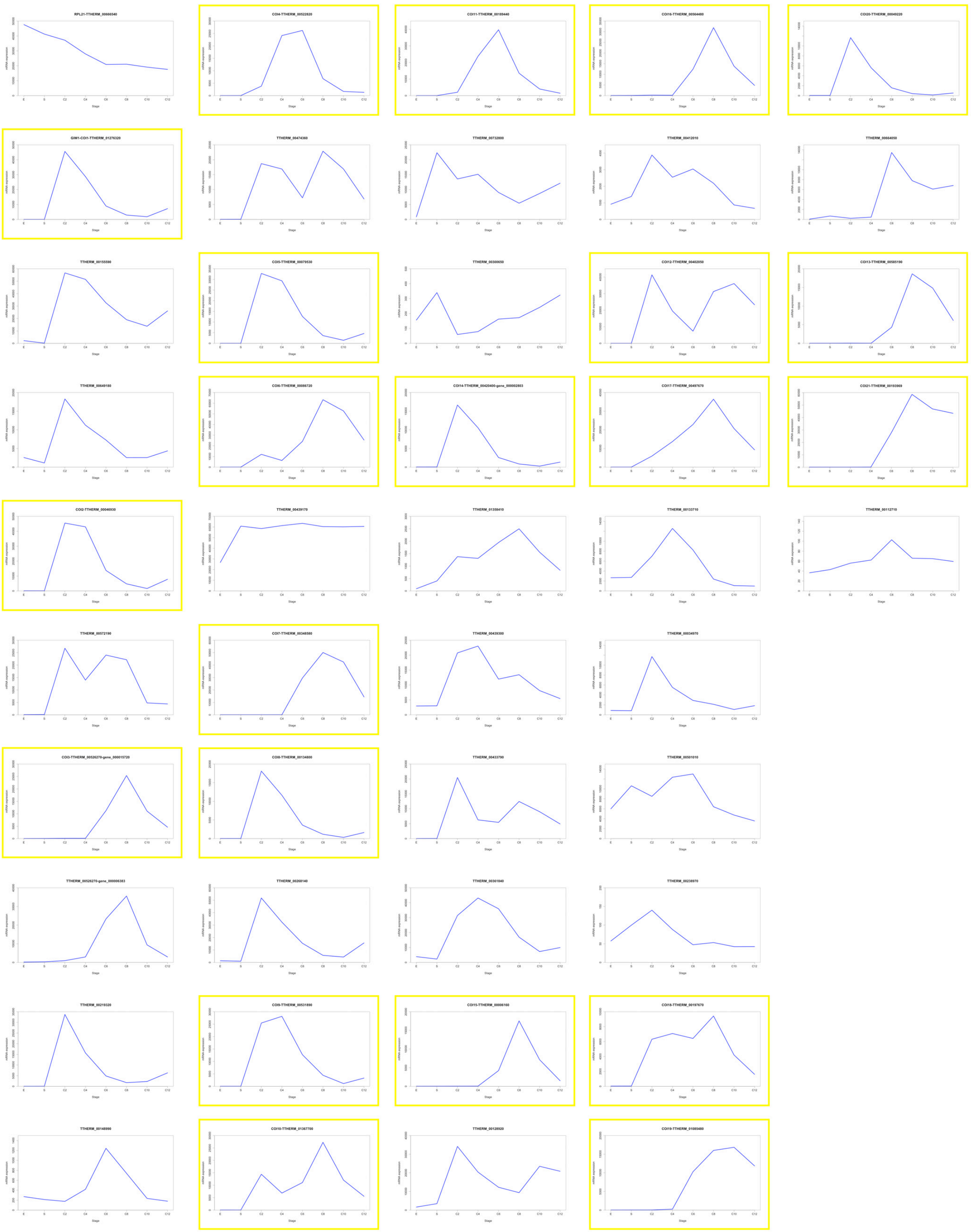

Figure S1. Gene expression profiles obtained by microarray-based analysis

The expression levels of mRNAs analyzed by microarray hybridization at different stages of the life cycle (Miao et al. 2009) were plotted. Exponentially (~3.5 x 105 cells/ml) growing cells (E); 24-hr starved cells (S); and conjugating cells at 2, 4, 6, 8, 10 and 12 hr post-mixing (C2, C4, C6, C8, C10 and C12, respectively) are indicated. The data were obtained from the Tetrahymena Functional Genomics Database (http://tfgd.ihb.ac.cn/). The COI genes are marked with yellow rectangles.

Figure S1

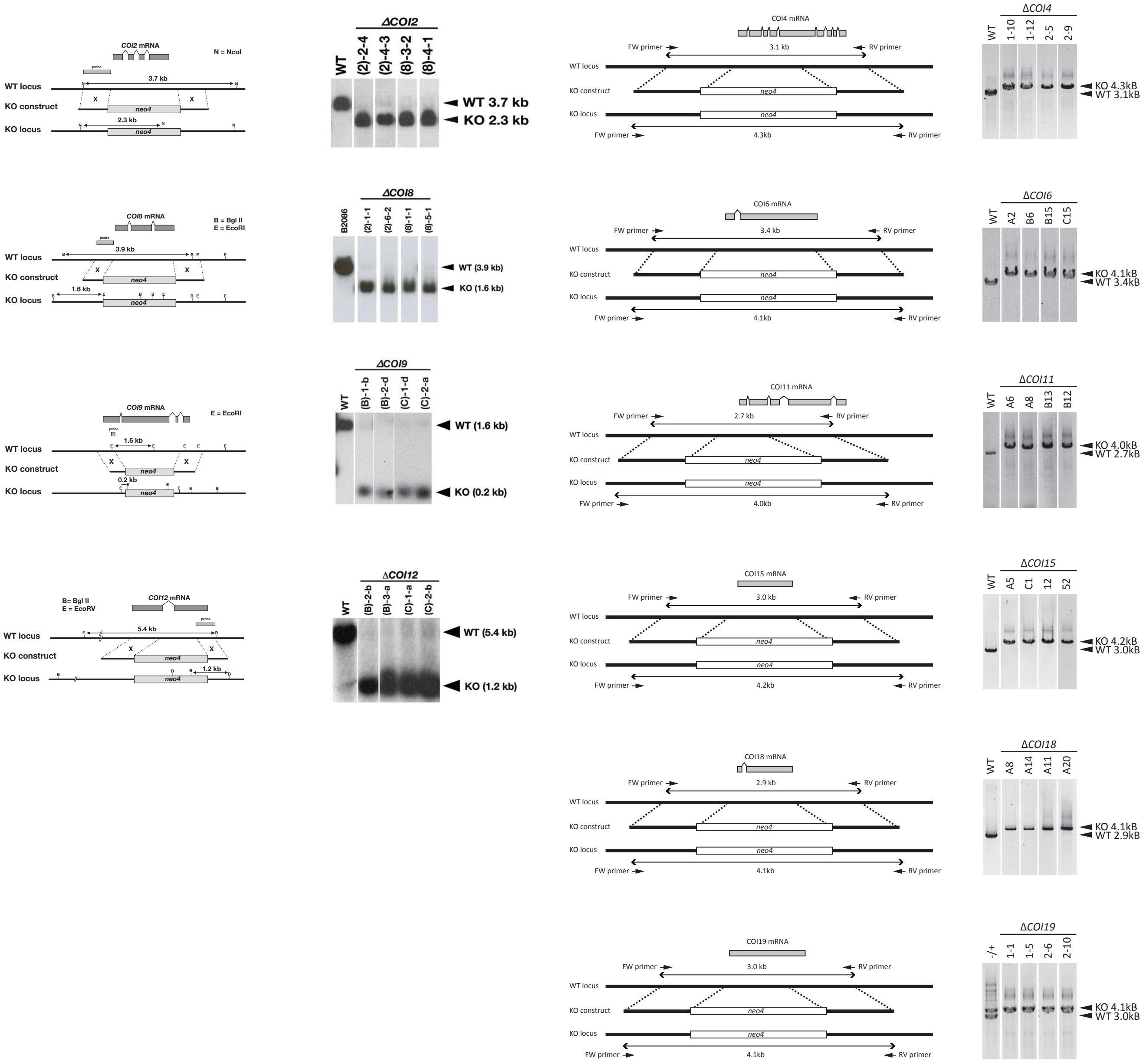

Figure S2. Construction of gene KO strains

Somatic (left) or germline (right) KO strains were produced by replacing parts or entire coding sequences with a drug-resistance cassette (neo4) by homologous recombination. For the somatic KO strains, complete replacements of the corresponding endogenous genes in the MACs were confirmed by Southern blot using the probes indicated. For the germline KO strains, homozygosity of the disrupted gene loci was confirmed by PCR using the primer sets shown as arrows in the schematic drawings.

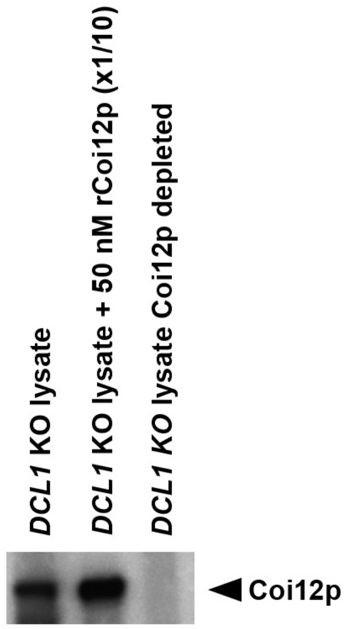

Figure S3. Comparison of endogenous and recombinant Coi12p

DCL1 KO cell lysate, 1/10th volume of DCL1 KO cell lysate mixed with 50 nM recombinant Coi12p (rCoi12p), and DCL1 KO cell lysate immunodepleted with Coi12p were analyzed by western blot using the anti-Coi12p antibody.

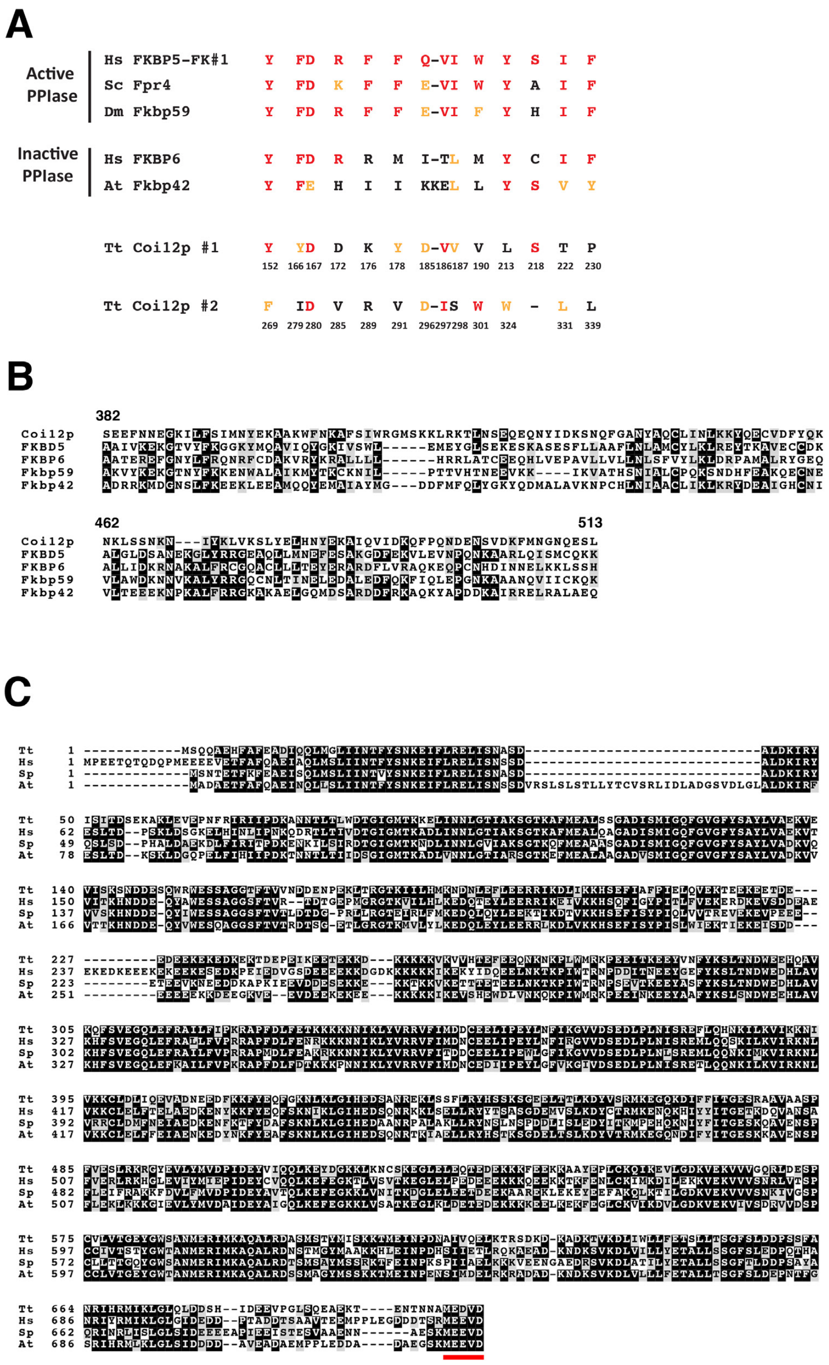

Figure S4. Sequence analyses of Coi12p and Hsp82p

(A) Conserved residues of the FK506-binding domains (FKBDs) of representative FK506-binding proteins and Coi12p were compared. The residues identical and similar to the first FKBD of human FKBP5 (top) are shown in red and orange, respectively. The numbers below the residues represent their positions in Coi12p. Hs: Homo sapiens, Sc: Saccharomyces cerevisiae, Dm: Drosophila melanogaster, At: Arabidopsis thaliana, and Tt: Tetrahymena thermophila.
(B) The TPR domains of representative FK506-binding proteins and Coi12p were compared. The numbers represent the positions of the corresponding residues in Coi12p.
(C) Hsp90 homologs in different eukaryotes were compared. The conserved C-terminal residues that are critical for their interaction with co-chaperones are marked with red lines.

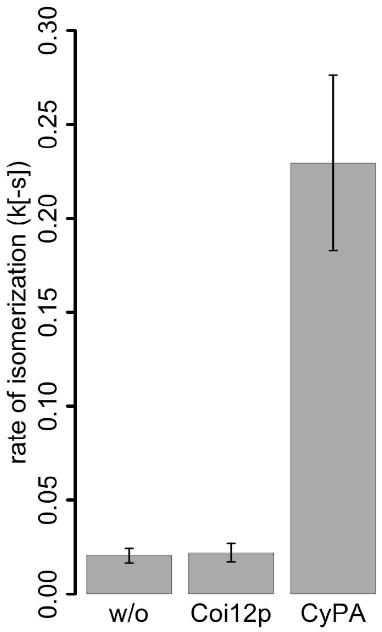

Figure S5. Peptidyl-prolyl isomerization assay with recombinant Coi12p

Peptidyl-prolyl isomerization activity was determined in a coupled assay with chymotrypsin. Cyclophilin A (CyPA) was used as a positive control. The buffer only condition (w/o) was used as a negative control. The standard deviation (SD) between technical replicates is indicated.

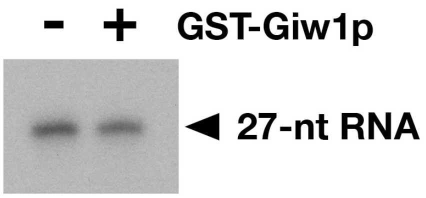

Figure S6. Stability of double-stranded 27-nt RNA incubated with GST-Giw1p

Radio-labeled double-stranded 27-nt RNAs were incubated with (+, 500 nM) or without (-) of GST-Giw1p in the buffer used for the loading assay for 1 hr. RNA was then purified from the reactions, separated in a denaturing gel and the radio-labeled 27-nt RNA was detected by autoradiography.

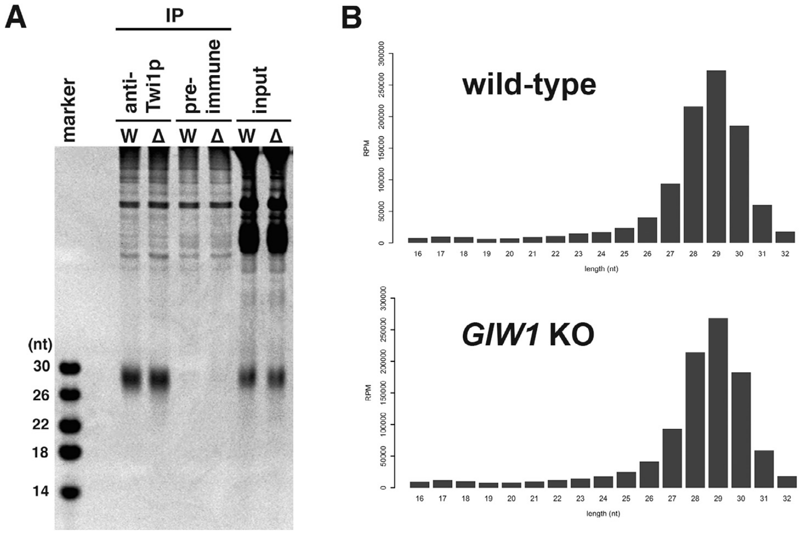

Figure S7. Twi1p-loaded scnRNAs in GIW1 KO cells

(A) Lysates from wild-type (W) and GIW1 KO (Δ) cells were prepared (input) and used for immunoprecipitation (IP) using either an anti-Twi1p antibody or a pre-immune rabbit serum. RNAs were extracted from input and immunoprecipitated samples, separated on a denaturing gel and visualized by the nucleic acid dye GelRed. The sizes of the RNA markers are indicated at the left. (B) Small RNAs (~16-32 nt) that co-precipitated with Twi1p from wild-type and GIW1 KO cells were analyzed by high-throughput sequencing. The size distributions of the sequenced small RNAs are shown.

Supplementary Table S1. Primers used for gene-expression analyses

Name	sequence (5’ -> 3’)
00006160FW	TGAAAATGAGGAAGACTATAGCC
00006160RV	TGTTAGAGCTAGGTTCTAATTCCATG
00034970FW	AAGGTATCTTCTTTGAATGACGCTG
00034970RV	GGAATGTTAAAGATGTTGAAGAGG
00046930FW	CTCTTAGAACTATGATTAAGTCA
00046930RV	TGCGACAGATAACTCTGCTATATGTC
00049220FW	GTAAGAAACAAGATGCAGCCATC
00049220RV	TACTCTAATAGGTAGAGCTGCTCAC
00079530FW	AAGTTGGTTTAAATCACTTCTGTCGTG
00079530RV	CTATGTATCTATGACCTGGATAGTGTC
00086720FW	AGAAGAATACAATGTTGAATA
00086720RV	TAAGCTTTACTAATTGTACGT
00112710FW	TTTATTACAGTAGAGTTGGAGG
00112710RV	TTTGAGCAAACTTCTCTAGAG
00128920FW	TTTCCACCAAGCCTAGGCAC
00128920RV	AGGAATTCTAAGAGGAATTGC
00133710FW	TCTTATTTGCGATTGCTGAACTAG
00133710RV	ACAACTTTGATAATGTTAAGGTC
00134800FW	CCTTCTGAAGATAAGAACTCATGTT
00134800RV	CCTTATGAATGCCTAATCCTCAC
00148990FW	GACTTTCATGATCAACTAAATGA
00148990RV	GGCATATTTGTATGGAATAACTG
00155590FW	CAATCTAAGTCTAAACATGCAGGTG
00155590RV	CTATCTTGAAACGACATGGTTGCTC
00189440FW	GCGATAGCATCAGTGAAGGAG
00189440RV	GAGACAAGTCAATGTCAGATAAGTGCTTGC
00193969FW	TTGAGCAGATGGATGATGAGTC
00193969RV	TCTTCTTTACCACTGTTTCAGG
00197670FW	TCCAGAGCAGCCATCTAGTG
00197670RV	AGCTTTCCTTAGTTAAGAATTCCTTG
00219320FW	AGGAGATATCAAAGAATCAAAATAAGTC
00219320RV	CTGTTTTTTGAGAGTCTAGTTATAAGC
00238970FW2	AGTAGCGAGTTCAAAATTGGTTGC
00238970RV	CATAAGAAGCTCCTACGTTATCGC
00268140FW	CTAAAACCATACAAAGTAATTGAGTGC
00268140RV	CAGCTATTGGAGTAGGTTATGAAG
00300650FW	GAATAATATAGAATACTTGCTAACTTATGG
00300650RV	AATTATAAAATCTTCAGTAGTTGAGAGG
00348580FW	CAAAGACATGGCTATCAAACTAG
00348580RV	GAGACTACTGATATCGAAGGTGC
00361840FW	ACCAATACAACTTTGCACAATTAACG
00361840RV	CACAGCTGCTTTCACTGACCAAC
00402050FW	CTTGATATGAGTTTTCTCTAAACTTGGC
00402050RV	CTCTAGACAAATATACCACTTTGCTG
00412010FW	GAGGATAAAGCAAAAGATCTCTTG
00412010RV	AGTTAGTATACTATTTGGTGG
00420400FW	TATGTATGTAAACTAAATAGAGAAGC
00420400RV	TCTGGATAATTCATCGCTGGTTTGC
00433790FW	TCGATTCACTTATCAAATTAGCAG
00433790RV	GTTGGATTAATTTCATAAGCTTTCAG
00439170FW	CTTACTCAGGTTGCTTGGCTTACTTAG
00439170RV	CCGAAGAATAAATCTTACACTGATTGTC
00439300FW	TAGGCGTCTAGAGAGAATTTGTG
00439300RV	TCCGTATTCGTACTGATCCTCATCATC
00474360FW	GTTGATGATTACTAAGACAACC
00474360RV	GATTCACACCTGCATGATGCATTGGCTC
00497670FW	ATGCCTCAAACTAGATTGCATGATG
00497670RV	TCGTCATATGGGCTTCTCCGAG
00501010FW	ATATTACTCTGCGCATGGATGC
00501010RV	TGAGACTGTCTTCTTCAATCCTCC
00522820FW	GAAAATGAGCTATTGAAGTAGCATC
00522820RV	GACATAATCATTTACTTCTCTATTTCAC
00526270FW	TGAAGACTTACTAGGTTAGCTC
00526270FW2	TGTTTCTCCTTCTACCATGCAGACTAGG
00526270RV	AATCCATTCTAAGACTCTACAG
00526270RV2	AGAATTTATTTAGCTCATCAACGCCAC
00531890FW	GTTTCAAATACTAAACTCCCAACATG
00531890RV	GATTCTGAATTTGTAATAGCGAAAGG
00564480FW	AATAGAAACAGATCTAGCCAGAG
00564480RV	TATTCTCTGCTTTATAGCTGTC
00572190FW	AGATACTTAGAAAGCTGAAGTAACAG
00572190RV	TCAATTAAAACGAGCAGGATATCAG
00585190FW	AGAACCTGATAGCTATTCTATGA
00585190RV	GACAATGGAGTGACTCCATTGCTTC
00649180FW	AAGCACGATAGCTACATCAAGG
00649180RV	TCATTAGGAGCAGTAGCAACAAC
00664050FW	ACCGAAGACAAACAACAGAGG
00664050RV	GGCATTTATCTTAACTTTTAGCCATC
00732800FW	CTTTTGGAAATAAGAGAAAGCAAGTG
00732800RV	ATTTATAGAGGAATGCCTTGTTGATG
01085480FW	AGAACTTATCAAAGCTCTTTAGC
01085480RV	TGAGTTGTAGTACGTTTACACC
01276320FW	AGCTCTTACTTAGGAACTATTGT
01276320RV	GAAATAGATGGAGTTAAGGTTC
01358410FW	TTCTGAAGATGAACCTAAGTGTTGC
01358410RV	CGAGGACATTTTCTAACTTAAAGTCG
01367700FW	AGCTATGACTAAATCATCACCAA
01367700RV	GTATCCAGATAGACCGAAATATGAGCAC
L21-FW	AAGTTGGTTATCAACTGTTGCGTT
L21-RV	GGGTCTTTCAAGGACGACGTA

Supplementary Table S2. Primers used to make KO constructs

Name	sequence (5′ -> 3′)
COI2_KO_5FW	AATAATTATTATGGAATCCATGG
COI2_KO_5RV	GTCTATCGAATTCCTGCAGCCCTTGAATTGAACAAAACACAC
COI2_KO_3FW	CTGGAAAAATGCAGCCCATTGATGGTTTGACCAAGC
COI2_KO_3RV	CATTTATTTGTAAGTGACTGAC
COI4-KO-3FW-SBHSN	TGCGGATCCGCTAGCACTAGTCATATGAAACGTTCAAGAGCCTCAAAAGAG
COI4-KO-3RV-SacII	CCGCCGCGGTAATTCACAAAATGGTTGGCATAC
COI4-KO-5FW-XhoI	GCGCTCGAGAGAATATTGATTAAAATTAAGCAGG
COI4-KO-5RV-SBHSN	ATGACTAGTGCTAGCGGATCCGCATGCTTAGAAGACACAACTTTCGTGCTC
COI6-KO-3FW2-SBHSN	TGCGGATCCGCTAGCACTAGTCATATGCTTATACAAGAATTCAGAAAGTGATAG
COI6-KO-3RV2-SacII	CCGCCGCGGTTATCAATTTTTATATAAGTAAAGGTTC
COI6-KO-5FW-XhoI	GCGCTCGAGACTGTAGCTATTAATAACTATTAG
COI6-KO-5RV2-SBHSN	ATGACTAGTGCTAGCGGATCCGCATGCGCTAAGTTTCAGCAAATTTTTAGATTC
COI8_KO_5FW	CATACAAAGCCTTTAAAACACTC
COI8_KO_5RV	GTCTATCGAATTCCTGCAGCCCAAGAAACACAAAATAAATCAATCTC
COI8_KO_3FW	CTGGAAAAATGCAGCCCTAACTGCTATGCAGAGTGTG
COI8_KO_3RV	ATAAATTAGCACACAAAAACAGC
COI9_KO_5FW	GTGGATCCGAGACACTAAATTAACTAGG
COI9_KO_5RV	CAGATCGTCGACAGATCTGGCACACGCTCTTAAGTAC
COI9_KO_3FW	TGTGCCAGATCTGTCGACGATCTGCTATTGACAAACGTAACT
COI9_KO_3RV	GGCTCGAGGCGGAATCAGAAATTGATCTGC
COI11-KO-3FW-SBHSN	TGCGGATCCGCTAGCACTAGTCATATGTCTATTTGATAATTGCAAGC
COI11-KO-3RV-SacII	CCGCCGCGGGCAAACATTAAAATGTCAAACTTAGC
COI11-KO-5FW-XhoI	GCGCTCGAGAAAAGATAGATGATATCTTTGGCTG
COI11-KO-5RV-SBHSN	ATGACTAGTGCTAGCGGATCCGCATGCTTATCCATTTTAATTTAAAAATAGGC
COI12_KO_5FW	GTGGATCCTAGAAGTTGTGTTGTAAGT
COI12_KO_5RV	TTCCAGGTCGACAGATCTGATAAGAGTAACTAGTTTGGTGC
COI12_KO_3FW	AGATCTGTCGACGACTTTCGAGAAAACTCATATCAAGCTTCAG
COI12_KO_3RV	GGCTCGAGTGTATGTATGAGTGTCTCTG
COI15-KO-3FW-SBHSN	TGCGGATCCGCTAGCACTAGTCATATGGCTAACCTTACTGTCAACGATATAT
COI15-KO-3RV-SacII	CCGCCGCGGTAACTCATTTGATTTTATTATTGGC
COI15-KO-5FW-XhoI	GCGCTCGAGTTTAGTCAATTCACCATTTTATTTTC
COI15-KO-5RV-SBHSN	ATGACTAGTGCTAGCGGATCCGCATGCGATTCTTAATTAATATCCTGATTGTG
COI18-KO-3FW-SBHSN	TGCGGATCCGCTAGCACTAGTCATATGGAAAAGAAGGTTTACAAATGAAAGTG
COI18-KO-3RV-SacII	CCGCCGCGGCAGATAATCCTCTTACTTCTTAAGAG
COI18-KO-5FW-XhoI	GCGCTCGAGCCTTAAATAGATAGAAAATAGACTG
COI18-KO-5RV-SBHSN	ATGACTAGTGCTAGCGGATCCGCATGCGAAGTAAATTAATTATAAAATTAAAGAG
COI19-KO-3FW-SBHSN	TGCGGATCCGCTAGCACTAGTCATATGTAAAATAAGAAAAAAGGTGTAAACG
COI19-KO-3RV-SacII	CCGCCGCGGACTATTGGATTTATATTTCAGTTC
COI19-KO-5FW-XhoI	GCGCTCGAGGGCTATTTTAATTAACATAGATTAG
COI19-KO-5RV-SBHSN	ATGACTAGTGCTAGCGGATCCGCATGCGAATCTTCTTCGTTTTATTTAACC

Supplementary Table S3. RNA oligos used for RNA loading assays

Name	sequence (5′ -> 3′)
sR-3hit-G	UCGAGUUGAUCUUUAGUUUCUUUUAGC
sR-3hit-aG	UAAAAGAAACUAAAGAUCAACUCGAUA
RNA23S	UCGAGUUGAUCUUUAGUUUCUUG
RNA23A	AGAAACUAAAGAUCAACUCGAUA
RNA27S	UCGAGUUGAUCUUUAGUUUCUUGUAGC
RNA27A	UACAAGAAACUAAAGAUCAACUCGAUA
RNA31S	UCGAGUUGAUCUUUAGUUUCUUGUAGCUUCC
RNA31A	AAGCUACAAGAAACUAAAGAUCAACUCGAUA
RNA35S	UCGAGUUGAUCUUUAGUUUCUUGUAGCUUCCAUUC
RNA35A	AUGGAAGCUACAAGAAACUAAAGAUCAACUCGAUA
