# Supplementary material for: A Tetrahymena Hsp90 co-chaperone promotes siRNA loading by ATP-dependent and ATP-independent mechanisms
Source: EMBO J. 2015 Jan 14;34(4):559–77. doi: 10.15252/embj.201490062 (PMC4331008; doi:10.15252/embj.201490062)
Supplement: Supplementary file 2 [file embj0034-0559-sd2.docx]

>COI12Ec

ATGGATTCATCGAATGAAAACACTCGCAGTTCTAGTTGTAATGAGCAGGAAACCAATTCGGAACTGCAGCAGTCCGTTAATGAATCATGTACGAGTCTGGAAGAACAGCAATTTTCCACCCCTTTCCTGTCATCCCAGGAGACAGTACAGGTCGATCAGGCATTTCAAGAATCCACCAAACAGAATGAAGGCACTTCTTCTTTTCTGAACGAAACCCAGTGCTACCAGGATTGCAAACCGAATCTGATTTCGAAAGGTGAGTCCAATCAACAGCAGTACAAAGAGCAGACCCAGGAAATTAGTGAAATTCTGGAAAATCCGTTCGCCCAAAACACACCTACGGACCAGTGGTCGATTGTTGAGGAAAAAGATGGAAATAAACAGGCCGATAGCTTTGATATTCTGCAGGATAAACTGATTAAAGAAGAAACCTACAATATTCCGTCTTGGAACTACGAACCGCAAGATTTCAAAGCAGATATTGACTTTGAGAAATATGACAAAGAGAACCTGGATCACCTGAAAAAAGTTTATAAACCGCCGCAGATCGAAGATGTCGTGGAAAAAGTCGAACAGGATGAAAATGGACTGAAAATTCAAACCCTGTCCCAGTTTAACATTCCAAAATGCAACATTCTGAAACTGGATATGAGTTTCCTGCAGACCTGGCAAAGCAAAGAAATCGTACCTTGTTTTACCATGTTTAGCGAACCTAAGACCAACAACGGTGAAAGCCTGAACTGCGATGAAGAAGAAACGAATCCGCAAATTGATAAAAAATGGTGCCGCGATGTTCAAGTCGTGTTCGAAGAGCGTGATCAGAACGGAGAACTGATTGACAAGTACCGTGTGGTAAAAGAAAAACGCACCGTCAAACTGCAGGGCGATATCTCGGCGAAATGGTATATTTGTCTGGAACAAATGAAACCAAATGAAACCAAATGGTTTAAGATGGAACAAAAACTGCTGTGGCAGATTGGCCAGAAAAACCTGGCGCAATATGAATGCATTTACCTGAAAATTCAGATCGTCGGTGTCAAAGCGCTGGCGCTGTCTGAGATGAAAGATAAACTGGAGAAAAATAATCTGCAGCCTCAGCAGCAAAATATCATTTATGAAGATTATAGCCAAATTGCACAAAAAAGTGAAGAATTCAACAATGAAGGCAAGATCCTGTTCTCCATTATGAACTATGAAAAAGCCGCCAAATGGTTTAATAAAGCGTTTTCGATTTGGCGTGGCATGTCCAAGAAGCTGCGCAAAACCCTGAACTCCGAACAAGAACAGAATTATATTGATAAAAGTAATCAATTCGGGGCAAACTACGCGCAGTGCCTGATTAATCTGAAGAAATACCAGGAATGCGTAGATTTTTATCAGAAAAATAAACTGAGTTCGAATAAAAATATCTACAAACTGGTTAAATCACTGTATGAACTGCATAACTACGAAAAGGCTATTCAGGTTATTGATAAACAGTTTCCACAGAACGACGAAAACAGCGTGGACAAATTTATGAATGGTAATCAGGAATCACTGCTGCCGGCGGAATTTCAAAAACTGAAGAGCCAGTGTCAGGTTAAGCTGAAAATGCTGGAAAATCAATCGATTAATACTTTTTATCGTAAACTGTTCCAGTAA

>HSP82Ec

ATGAGCCAACAAGCGGAACATTTCGCATTTGAAGCCGACATCCAACAACTGATGGGTCTGATTATTAACACCTTTTACAGCAACAAAGAAATCTTTCTGCGTGAACTGATTAGTAATGCGTCCGATGCCCTGGACAAAATTCGCTATATTAGCATCACCGATTCTGAAAAAGCAAAACTGGAAGTTGAACCGAACTTCCGTATCCGCATTATCCCGGATAAAGCTAACAATACCCTGACGCTGTGGGACACCGGCATTGGTATGACGAAAAAAGAACTGATCAACAATCTGGGCACCATTGCGAAAAGTGGTACGAAAGCATTTATGGAAGCTCTGAGCTCTGGCGCCGATATTAGCATGATCGGCCAGTTTGGCGTTGGTTTCTATTCTGCGTACCTGGTCGCCGAAAAAGTGGAAGTTATCTCAAAATCGAACGATGACGAAAGCCAATGGCGTTGGGAAAGTTCCGCAGGCGGTACCTTTACGGTGGTTAACGATGACGAAAATCCGGAAAAACTGACCCGCGGCACGAAAATCATCCTGCATATGAAAAACGATAACCTGGAATTCCTGGAAGAACGTCGCATTAAAGACCTGATCAAAAAACACAGCGAATTTATTGCCTTCCCGATCGAACTGCAGGTGGAAAAAACCGAAGAAAAAGAAGAAACGGATGAAGAAGACGAAGAGAAAGAAAAAGAAGATAAAGAAAAAACCGACGAACCGGAAATCAAAGAAGAAACGGAGAAAAAAGATAAGAAAAAGAAAAAAGTCAAAGTCGTGCATACCGAATTCGAAGAACAGAACAAAAACAAACCGCTGTGGATGCGTAAACCGGAAGAAATCACCAAAGAAGAATACGTGAACTTCTACAAAAGCCTGACGAATGATTGGGAAGAACACCAGGCGGTCAAACAATTCTCTGTGGAAGGCCAACTGGAATTTCGTGCAATTCTGTTCATCCCGAAACGCGCTCCGTTTGACCTGTTTGAAACCAAGAAAAAGAAAAACAACATCAAACTGTACGTTCGTCGCGTCTTTATTATGGATGACTGCGAAGAACTGATCCCGGAATACCTGAATTTCATTAAAGGTGTTGTCGATTCAGAAGACCTGCCGCTGAACATTTCGCGCGAATTCCTGCAGCATAACAAAATCCTGAAAGTGATTAAGAAAAACATTGTTAAAAAATGTCTGGATCTGATCCAGGAAGTTGCGGATAACGAAGAAGACTTCAAAAAATTCTACGAACAATTTGGCAAAAATCTGAAACTGGGTATTCATGAAGATTCGGCCAACCGTGAAAAACTGTCATCGTTCCTGCGCTATCACAGCTCTAAAAGTGGCGAAGAACTGACCACGCTGAAAGATTACGTTTCCCGTATGAAAGAAGGCCAGAAAGACATTTTCTTTATCACCGGTGAATCTCGCGCGGCAGTCGCAGCTAGTCCGTTTGTGGAATCCCTGCGTAAACGCGGTTATGAAGTGCTGTACATGGTTGATCCGATCGACGAATATGTGATTCAGCAACTGAAAGAATACGATGGCAAAAAACTGAAAAACTGCTCCAAAGAAGGTCTGGAACTGGAACAGACCGAAGATGAAAAGAAAAAATTCGAAGAGAAAAAAGCGGCCTATGAACCGCTGTGCAAACAGATTAAAGAAGTTCTGGGCGATAAAGTCGAAAAAGTGGTTGTCGGTCAACGTCTGGACGAATCACCGTGTGTCCTGGTGACCGGCGAATATGGTTGGTCGGCGAACATGGAACGTATCATGAAAGCACAGGCTCTGCGCGATGCCAGCATGTCTACCTACATGATTAGCAAGAAAACCATGGAAATCAACCCGGACAATGCAATTGTGCAAGAACTGAAAACCCGCTCCGATAAAGACAAAGCTGATAAAACGGTTAAAGACCTGATCTGGCTGCTGTTTGAAACCTCACTGCTGACGTCAGGTTTTTCGCTGGATGACCCGAGTTCCTTCGCGAACCGTATCCATCGCATGATTAAACTGGGCCTGCAGCTGGATGACTCTCACATTGATGAAGAAGTGCCGGGTCTGTCGCAGGAAGCGGAAAAAACCGAAAATACCAACAACGCAATGGAAGATGTGGACTGA

>pBMNB1-HA

GTGGCACTTTTCGGGGAAATGTGCGCGGAACCCCTATTTGTTTATTTTTCTAAATACATTCAAATATGTATCCGCTCATGAGACAATAACCCTGATAAATGCTTCAATAATATTGAAAAAGGAAGAGTATGAGTATTCAACATTTCCGTGTCGCCCTTATTCCCTTTTTTGCGGCATTTTGCCTTCCTGTTTTTGCTCACCCAGAAACGCTGGTGAAAGTAAAAGATGCTGAAGATCAGTTGGGTGCACGAGTGGGTTACATCGAACTGGATCTCAACAGCGGTAAGATCCTTGAGAGTTTTCGCCCCGAAGAACGTTTTCCAATGATGAGCACTTTTAAAGTTCTGCTATGTGGCGCGGTATTATCCCGTATTGACGCCGGGCAAGAGCAACTCGGTCGCCGCATACACTATTCTCAGAATGACTTGGTTGAGTACTCACCAGTCACAGAAAAGCATCTTACGGATGGCATGACAGTAAGAGAATTATGCAGTGCTGCCATAACCATGAGTGATAACACTGCGGCCAACTTACTTCTGACAACGATCGGAGGACCGAAGGAGCTAACCGCTTTTTTGCACAACATGGGGGATCATGTAACTCGCCTTGATCGTTGGGAACCGGAGCTGAATGAAGCCATACCAAACGACGAGCGTGACACCACGATGCCTGTAGCAATGGCAACAACGTTGCGCAAACTATTAACTGGCGAACTACTTACTCTAGCTTCCCGGCAACAATTAATAGACTGGATGGAGGCGGATAAAGTTGCAGGACCACTTCTGCGCTCGGCCCTTCCGGCTGGCTGGTTTATTGCTGATAAATCTGGAGCCGGTGAGCGTGGGTCTCGCGGTATCATTGCAGCACTGGGGCCAGATGGTAAGCCCTCCCGTATCGTAGTTATCTACACGACGGGGAGTCAGGCAACTATGGATGAACGAAATAGACAGATCGCTGAGATAGGTGCCTCACTGATTAAGCATTGGTAACTGTCAGACCAAGTTTACTCATATATACTTTAGATTGATTTAAAACTTCATTTTTAATTTAAAAGGATCTAGGTGAAGATCCTTTTTGATAATCTCATGACCAAAATCCCTTAACGTGAGTTTTCGTTCCACTGAGCGTCAGACCCCGTAGAAAAGATCAAAGGATCTTCTTGAGATCCTTTTTTTCTGCGCGTAATCTGCTGCTTGCAAACAAAAAAACCACCGCTACCAGCGGTGGTTTGTTTGCCGGATCAAGAGCTACCAACTCTTTTTCCGAAGGTAACTGGCTTCAGCAGAGCGCAGATACCAAATACTGTCCTTCTAGTGTAGCCGTAGTTAGGCCACCACTTCAAGAACTCTGTAGCACCGCCTACATACCTCGCTCTGCTAATCCTGTTACCAGTGGCTGCTGCCAGTGGCGATAAGTCGTGTCTTACCGGGTTGGACTCAAGACGATAGTTACCGGATAAGGCGCAGCGGTCGGGCTGAACGGGGGGTTCGTGCACACAGCCCAGCTTGGAGCGAACGACCTACACCGAACTGAGATACCTACAGCGTGAGCTATGAGAAAGCGCCACGCTTCCCGAAGGGAGAAAGGCGGACAGGTATCCGGTAAGCGGCAGGGTCGGAACAGGAGAGCGCACGAGGGAGCTTCCAGGGGGAAACGCCTGGTATCTTTATAGTCCTGTCGGGTTTCGCCACCTCTGACTTGAGCGTCGATTTTTGTGATGCTCGTCAGGGGGGCGGAGCCTATGGAAAAACGCCAGCAACGCGGCCTTTTTACGGTTCCTGGCCTTTTGCTGGCCTTTTGCTCACATGTTCTTTCCTGCGTTATCCCCTGATTCTGTGGATAACCGTATTACCGCCTTTGAGTGAGCTGATACCGCTCGCCGCAGCCGAACGACCGAGCGCAGCGAGTCAGTGAGCGAGGAAGCGGAAGAGCGCCCAATACGCAAACCGCCTCTCCCCGCGCGTTGGCCGATTCATTAATGCAGCTGGCACGACAGGTTTCCCGACTGGAAAGCGGGCAGTGAGCGCAACGCAATTAATGTGAGTTAGCTCACTCATTAGGCACCCCAGGCTTTACACTTTATGCTTCCGGCTCGTATGTTGTGTGGAATTGTGAGCGGATAACAATTTCACACAGGAAACAGCTATGACCATGATTACGCCAAGCGCGCAATTAACCCTCACTAAAGGGAACAAAAGCTGGAGCTCCACCGCGGTGGCGGCCGCTCGAGTCTAGAGTTGTTTGGATAATTAGATCTCTCTCTTTCTATCGTATTTTGCAATAATAGGTATTAACTTTTATACTGATTGTTAGTAGATGCCTTCAAATTTTCTTTTTATTTAAATTCACATGCTATATCTTTTAAAACACTCCACATTTTATTGTTGCTAACTGTGCTATTGATCTTTAAGTCAATAGCTGCTCATTTTGTTGAACTCCACAGAGACACTAAATTTGTTTATTTTGATGGATGCTTTATAATTAAAGTTACGTAATCTGCTTGACATTTAGCCAACTATATAAAAAAGATCAAAATGTAGCTTAAATCTCAAAAAATCATCATAATTTACTATCAAATTATTAAGAAATTCATATAATCACCACTTTATTGACTTTTATTCATCTTATAGAGTGATAGTAGAGTTGAGCCAAATTGATACTTGTTTACGTTGTATTATTTTGAAATTTTAAAAAAATGAAAATGAGAGAAAAATTTATTTAAATTTGAGCTTAGAATCTTTAAGGAAGATCAAAAATGGGCTAACTAAATGTTAGAGTACGAAGACTGTTCTTGAAATAAAGTGTCATCCTTTACGAATCAAGTTGCTACTTTAATGAATAATAGAATTTGAGGTAGAGCTAAAATGAGAGATATAGTAATGCTATTGGATTATATTTGGTTTGTATGATGGTTTTTCTTTGGTAAATGAATGATATAAATGAAGAGTGGCAATAAAATTAATTGAAATTGAATGAAAAAATGAATAGAAATTAAAGAAGAGTATAATTTTATTTTTGAATTTTATTTAAATTTTAATGCGTGTATTTATTTGGGTGATGTCGACTTGATATCTTCAAAGTATGGATTAATTATTTCAAATTATTAGAAGGTAATTAATCTGCATAAATTCAAAACTATAAAAATAAAACATTAAAATTAATTCAACCTTATTGAAGCATCAAAATCTGAATCTCTAGAAAGACTGATTCTGATTGGATAATTTTTCGGCGCTAAGGATTTTGGATTAAAGAAAATTAGATTTAATTATTAATCATGATTTGAATAGGATAGCAAGAATATTTGTTTGGTTTAAAAGGGAAAGCGGGTAATTATCAAAAATTTATAAATAATTTTAAAACAATAAATAGAAAAACAAATAAGATTATAAAAACTTACAAAAATGATTGAACAAGATGGTTTACACGCTGGTTCTCCCGCCGCTTGGGTCGAAAGACTTTTCGGTTATGACTGGGCTCAACAAACCATCGGTTGCTCTGATGCCGCCGTCTTCCGTCTTTCTGCTCAAGGTCGTCCTGTTCTTTTCGTCAAGACCGACCTTTCTGGTGCCCTTAATGAACTTCAAGATGAAGCTGCCCGTCTTTCTTGGCTTGCCACCACCGGTGTTCCTTGCGCTGCTGTCCTTGACGTTGTCACTGAAGCCGGTAGAGACTGGCTTCTTTTAGGTGAAGTCCCCGGTCGAGATCTTCTTTCTTCTCACCTTGCTCCTGCCGAAAAAGTTTCTATCATGGCTGATGCTATGCGTCGTCTTCATACCCTTGATCCCGCTACCTGCCCTTTCGACCACCAAGCCAAACATCGTATCGAACGTGCTCGTACTCGTATGGAAGCCGGTCTTGTCGATCAAGATGATCTTGACGAAGAACATCAAGGTCTTGCCCCTGCCGAACTTTTCGCCAGACTTAAGGCCCGTATGCCCGACGGTGAAGATCTTGTCGTCACCCATGGTGATGCCTGCTTACCCAATATCATGGTTGAAAATGGTCGTTTTTCTGGTTTCATCGACTGTGGTCGTCTTGGTGTCGCCGACCGTTATCAAGATATTGCCTTAGCTACCCGTGATATCGCTGAAGAACTTGGTGGTGAATGGGCTGACCGTTTCCTTGTCCTTTACGGTATCGCCGCTCCCGATTCTCAACGTATCGCCTTCTATCGTCTTCTTGACGAATTCTTCTGAGATCCTTAAATTAAAAATTCAATATATATTTACAAACTTTCATATAAAATAAATATATTATATAAAATTAATTTTTAGTGTATTATATTAACATTAAAGCACCAAAAAAACGTGTTAATATACTACTATAAAATATAATTTATTCCAAATTGACTAAAATCATTATTTTACAACTCATTTGTATATATATTTTATGTCAATTATTTTTTTTAACTTTCTAAAAAAAAAAATTCCTCTTCACATACATGTTAGCTCTTAAAAATTTGTCTGCAAATCCAATAATAATATTTTTTTTTTGCCATTAAATTTTCAAATTTTTACTGGAAAAATGCAGCCCGGGGGATCAGACAATTTATTTCTAAAAAATATTTAAAAATAAAAAATAATAAGGGTTTTGAATAACTCCTTTAATTTAAATACACATTTTTAAATTTTTTTTAGCTCTTTAAATATTCATAAAAATAAAAAATAACTAACTAAAAATAAATAAAAAGATAATAATGATTAAAGGTATAATACTGTATAAGAAAAAACATAATAGAGTACTTATTTTTTATATCACTATTTTTAATATCTTGAAAGCAAAACTTTTTTATATATCTTAAAATATATTGTATCGTTTATTCAATTATTTTCTTTAAATTTCAAATATATTGATAAAAAAGATGACATGTTTTTTAAAGAAAACATGAAATATAAAATAGATAAATATCAATTATTTTATTTATTAAATATATAAGCTGCTCAAAACATAGCTCATTCATCAATTATAATATGTGAATCATTAATTTTCAAAATATTACTCATTATTTAGGCTATCATTTATTTTTTATTTTCAATTATCCGTTTCTATTATATTTTAATATTAAGTTGTGATTCTTGAATTTTGTGTCATGAATTATTTGTAAATCTTTTTATTTCTGATAAAAAATACAAATTGATTGACTCATGATTTAAATCATGAGTCAACCTAACTAATTTTCAAAATTCTTCTATTCTAAAATATAGATGTGATTCTTGAATCTCTCTTGAATATAAAGTAATTTTTTATATTTCTGATATAATTCTTAGCTACGTGATTCACGATTTATGCAATGATCCATATAAAATAATGTAAATAGTGTATATATATATATTCGTCTTTTTTATTCTTTATATAATTTAAAAAAATTAAAAAAATTTAATAAAGCTCTAATAAAATAAATAATAATACTAAACTTAAACATATGGGATCATATCCTTATGATGTTCCTGATTATGCTGGATCCGCTAGCACTAGTTGAGCGAACTGAATCGGTCAGCTAAACCAACCAATCAACATAATAAACTTTATTATTTTTACTTAAGCATCTTACTGTTGTTGTAATAGTAGAGAAAGAAATACCCAATTAACTTCATTCACATAACATTAATATCTATAAACATCTTTTTTCTCACATATATACAACTCTCTAAATCAACAAATAACTTTTTAAAAATAATGGATATATATTAACAAAATAATATATCTCTTTTTACAAAATAGTTCTTATATAAATACGTATTCTGCACTCACCCGCATTTTTCACAACAAAAACATACCAAAAAAATTCTTACTTCTACATGTTTCCTTTCTTATTATTACAAAATTATTTTATAAATAGCATACAAAAATAAATACAATAAAAAAATAAACAAAATCCTTTTTTATTTTGAATTATTTAAAACAAATATTTTCAATCAATCAGTCAGTCAGCATAATATTAAAGCAACAAAACAAACCCAAGTTGTTTTTATAGTTTTTTAATTGCTTTTCAGTACTATAAATAAATTTGTTATTACTTCAAGATTGATAAACTTCTTTTTTAAATTAAATATCTATGAATGAATAAATAAGTTGATATCTCTTTTTAACTTGTTTTCCTCTCTTTTACTTACTTGCCAATTTTTTTTTTAAATTAAAGAAATATCTTTTTATTTTTCAAAAACAAAATTTATTTTCCCTTGTATACAAAAACCCCTTTATTTAAATAAAATCTTTATGCCCATCAATAGCCACATCTTCTCGAGGGGGGGCCCGGTACCCAATTCGCCCTATAGTGAGTCGTATTACGCGCGCTCACTGGCCGTCGTTTTACAACGTCGTGACTGGGAAAACCCTGGCGTTACCCAACTTAATCGCCTTGCAGCACATCCCCCTTTCGCCAGCTGGCGTAATAGCGAAGAGGCCCGCACCGATCGCCCTTCCCAACAGTTGCGCAGCCTGAATGGCGAATGGGACGCGCCCTGTAGCGGCGCATTAAGCGCGGCGGGTGTGGTGGTTACGCGCAGCGTGACCGCTACACTTGCCAGCGCCCTAGCGCCCGCTCCTTTCGCTTTCTTCCCTTCCTTTCTCGCCACGTTCGCCGGCTTTCCCCGTCAAGCTCTAAATCGGGGGCTCCCTTTAGGGTTCCGATTTAGTGCTTTACGGCACCTCGACCCCAAAAAACTTGATTAGGGTGATGGTTCACGTAGTGGGCCATCGCCCTGATAGACGGTTTTTCGCCCTTTGACGTTGGAGTCCACGTTCTTTAATAGTGGACTCTTGTTCCAAACTGGAACAACACTCAACCCTATCTCGGTCTATTCTTTTGATTTATAAGGGATTTTGCCGATTTCGGCCTATTGGTTAAAAAATGAGCTGATTTAACAAAAATTTAACGCGAATTTTAACAAAATATTAACGCTTACAATTTAG
