# Supplementary material for: A Tetrahymena Hsp90 co-chaperone promotes siRNA loading by ATP-dependent and ATP-independent mechanisms
Source: EMBO J. 2015 Jan 14;34(4):559–77. doi: 10.15252/embj.201490062 (PMC4331008; doi:10.15252/embj.201490062)
Supplement: Supplementary file 3 [file embj0034-0559-sd3.pdf]

THE
EMBO
JOURNAL

Manuscript EMBO-2014-90062

A *Tetrahymena* Hsp90 co-chaperone promotes siRNA loading by ATP-dependent and ATP-independent mechanisms

Sophie Woehrer, Lucia Aronica, Jan H.Suhren, Clara Jana-Lui Busch, Tomoko Noto and Kazufumi Mochizuki

Corresponding author: Kazufumi Mochizuki, IMBA

Review timeline:

Submission date:	15 September 2014
Editorial Decision:	15 October 2014
Revision received:	30 November 2014
Accepted:	08 December 2014

Editors: Alexander Kohlmaier and Hartmut Vodermaier

Transaction Report:

(Note: With the exception of the correction of typographical or spelling errors that could be a source of ambiguity, letters and reports are not edited. The original formatting of letters and referee reports may not be reflected in this compilation.)

1st Editorial Decision 15 October 2014

Thank you for submitting your manuscript "A Tetrahymena Hsp90 co-chaperone promotes siRNA loading by ATP-dependent and ATP-independent mechanisms" for our editorial consideration. We have now received the comments of three expert referees, which you will find copied below.

As you will see, all the referees consider your findings interesting and significant, rate the technical quality of your experiments high, and, overall, support publication of your manuscript pending revision of a few remaining points. The EMBO Journal invites you, therefore, to submit a revised version of your manuscript. We think that addressing the points raised by the referees will be straight-forward, and we also consider the relevant suggestions made by the referees to address these specific concerns very constructive.

I will not repeat here in detail all the referees' specific requests but highlight only some key points relating to mechanistic clarifications that we consider particularly important: (yet, please keep in mind that it is our policy to allow only a single round of major revision and that it is therefore important to carefully respond to all points in your point-to-point response).

a. As indicated in referee #2's major point 3, additional supporting evidence to substantiate the lid model, i.e. that Giw1p restrains loading of scnRNAs into Twi1p, could make the manuscript stronger overall.

© European Molecular Biology Organization

b. We also think that some additional experimentation would be helpful to more decisively corroborate the functional loading of siRNAs bound to Twi1p into RISC, and whether Twi1p-associated siRNAs are double- or single stranded (in the absence of ATP/the chaperone machinery). This could be addressed by IPs, standard biochemical tests and measurements of strand separation kinetics, as most clearly spelled out by referee #2's major points1-2, but also highlighted to some extent by referees 1 and 3.
c. We would also please ask you not to report on "data not shown" in the manuscript, but to include this type of information directly in the manuscript or the supplementary information.

Together, we would be very pleased if you agreed to invest the necessary time and effort to address the specific points raised by the referees during your revision and I am very positive about the outcome of your revision, given the promising remarks made already now by the referees.

Congratulations to your work already at this point. I am looking forward to receiving your revised manuscript.

Referee #1:

The authors identified conjugation induced genes (COI) and show that one of them COI12 is an essential factor for loading small RNAs into the Argonaute Twi1 in vitro and in vivo. COI12 is orthologous to the Hsp90 co-chaperone Shutdown or Fkbp6 that act in the piRNA pathway. Surprisingly, they clearly demonstrate that although the protein has an ATP-dependent and Hsp90-dependent activity, it also has ATP-independent and Hsp90-independent role in small RNA loading into the Argonaute. They propose that this activity is mediated by counteracting the role of the Ago-interacting protein Giw1p. The experiments are clear and nicely executed. I support its publication.

Minor remarks:
1) Coi12p does not interact with small RNA duplex (data not shown). Can the authors comment on the possibility that the recombinant protein made in E. coli is unable to bind RNA because of lack of post-translational modifications.
2) In the Coi12 KO, are precursors of the scan RNAs still present? (in Fig. 3F, G).
3) Figure 4C: Do the authors know the identity of the smaller, fainter bands recognized by the Coi12p antibody? These seem to be specific as they are removed by the anti-Coi12p immunoprecipitation.
4) Figure S5: Did the authors examine whether the Coi12 bound Fk506? Examination of isomerase activity is fine, but it is possible that the target peptide might not be the optimal one. Given the non-TPR-mediated function (non-ATP and non-Hsp90) action of Coi12, this has to be considered. Perhaps the authors might want to comment on this in the discussion.

Referee #2:

In this manuscript, Mochizuki and colleagues report that the Tetrahymena Hsp90 co-chaperone Coi12p promotes loading of siRNAs into the Argonaute protein Twi1p, which is required for programmed DNA elimination. Coi12p-mediated siRNA loading was promoted by ATP in a manner dependent on the TPR domain of Coi12p and Hsp90. However, in the absence of ATP, Hsp90 and/or the TPR domain of Coi12p, Twi1p could still bind to a considerable amount of siRNAs. Moreover, when the Twi1p-binding protein Giw1p was knocked out, ATP was no more required for Twi1p to bind to siRNAs. Accordingly, the authors propose that Twi1p acts as a "lid" that inhibits loading of siRNAs into Twi1p, which may be displaced by the chaperone machinery to promote loading. Many of the experiments are appropriately performed and the proposed model is novel and interesting. However, the following points should be addressed before the manuscript is published in The EMBO Journal.

Major points:

1. Immunoprecipitation is a convenient method to check "binding" between Twi1p and siRNAs. However, it remains unknown if those siRNAs bound to Twi1p in the absence of ATP or the chaperone machinery have really been "loaded" in a canonical manner and produced functional RISC. It is formally possible that, in the absence of ATP or the chaperone machinery, siRNAs are aberrantly bound to Twi1p without efficient RISC formation. Indeed, the in vivo phenotype of Coi12KO + dTPR is extremely severe (Fig. 6), compared to the modest inhibition of siRNA binding in the absence of the TPR domain, Hsp90 or ATP in vitro (Fig. 5). The authors should check the double-stranded vs single-stranded states of those Twi1p-coIPed siRNAs in the presence or absence of ATP and show that the two strands are separated in the same kinetics. Measuring and quantitatively comparing their target-slicing activities (normalized to the amount of bound siRNAs) will also be informative.
2. Similarly, the authors should check those siRNAs bound to Twi1p in Gwi1KO lysate (Fig. 7) have produced functional RISC.
3. It is critical for the authors to validate their "lid model" in vivo. The authors should immunoprecipitate Twi1p from Coi12p and Giw1p double-KO cells and Coi12p single-KO cells, and show that siRNAs are more efficiently loaded into Twi1p in the absence of Giw1p.

Minor points:

1. The authors should confirm that the same amount of Twi1p was IPed in the presence or absence of ATPgammaS in Fig. 5E, as well as in other in vitro IP experiments.
2. The amount of input siRNAs in Fig. 7D should be shown to exclude the possibility that siRNAs were simply degraded by some contaminants in GST-Giw1p.
3. Does Giw1p bind to small RNAs? Is it possible that Twi1p and Giw1p compete for siRNA binding?
4. Fig. 7E requires a positive control of wild-type Twi1p, which should predominantly produce ssRNAs.
5. Page 17, line 9: Iki et al., 2010, 2012 in fact used tobacco AGO1, not Arabidopsis AGO1.
6. Fig. 5 legend: His-Coi12p-ΔC should read His-Hsp82p-ΔC.
7. The text is sometimes overstated and should be carefully toned down. For example, given the strong in vivo phenotype of Coi12KO+dTPR (Fig. 7), it is too strong to state "Hsp90- and ATP-independent loading of siRNA occurs in vivo and is an integral part of the DNA elimination process in Tetrahymena." Moreover, it remains unknown if their findings are applicable to other species.

Referee #3:

The authors of this manuscript identify a protein, Coi12p, that is expressed exclusively during Tetrahymena sexual reproduction and is required to load the argonaute protein Twi1p prior for its functions in genome remodeling. Using a careful and thorough combination of experiments performed in vivo and in extract, the authors show that Coi12p specifically participates in loading sRNAs into Twi1p. They then investigate the molecular mechanism of Coi12p-dependent sRNA loading, and demonstrate that Coi12p loads Twi1p via both an ATP-dependent and ATP-independent pathway, though the ATP-dependent pathway predominates in vivo. The authors then demonstrate that the ATP-dependent activity of Coi12p relieves the inhibition of Giw1p, which has previously been shown to directly associate with Twi1p and is required for proper localization of Twi1p during sexual reproduction.

Overall, the experiments in this paper are exquisitely conducted and controlled, and the conclusions are well-supported by the experimental data reported. The finding that an Argonaute protein requires a directly associating factor to limit its promiscuity in associating with sRNAs and that relieving this inhibition is a key step in sRNA loading is both novel and provocative. However, the manuscript could be improved by more thoroughly discussing the relationship between the functions of Giw1p identified in this manuscript and those previously published in Noto, et al. Giw1p has previously been shown to associate preferentially with molecules of Twi1p loaded with single-stranded sRNAs, while the model presented in this manuscript requires Giw1p association with unloaded Twi1p. Reconciling these findings with one another in the discussion would be appreciated.

1st Revision - authors' response 30 November 2014

Responses to the referees' comments:

Referee #1:

1) Coi12p does not interact with small RNA duplex (data not shown). Can the authors comment on the possibility that the recombinant protein made in E. coli is unable to bind RNA because of lack of post-translational modifications.

We agree with the referee's comment and have added the following descriptions (underlined) to Discussion:
"Coi12p is unlikely to be such a scnRNA duplex-binding protein because our attempts to detect a direct interaction between recombinant Coi12p and scnRNA duplexes in vitro have failed (data not shown), although the recombinant Coi12p expressed in *E. coli* might lack some post-translational modification critical for its binding to scnRNA duplexes."

2) In the Coi12 KO, are precursors of the scan RNAs still present? (in Fig. 3F, G).

Because scnRNAs were detected in *COI12* KO cells, we believe that their precursors are also produced normally in these cells. However, because Dcl1p, the Dicer enzyme that processes the precursors to scnRNAs, is still present in *COI12* KO cells, we believe the quick processing of the precursors by Dcl1p makes detection of the precursors very hard in *COI12* KO cells (as well as in wild-type cells).

3) Figure 4C: Do the authors know the identity of the smaller, fainter bands recognized by the Coi12p antibody? These seem to be specific as they are removed by the anti-Coi12p immunoprecipitation.

We believe that these smaller bands were caused by some partial degradation of Coi12p during the course of the preparation of cell lysates, which takes ~30 min after opening cells. This view is supported by the fact that the same antibody did not detect these smaller bands in the western blot when using total proteins (Figure 2A), which were prepared by quick inactivation of proteases by TCA precipitation.

4) Figure S5: Did the authors examine whether the Coi12 bound Fk506? Examination of isomerase activity is fine, but it is possible that the target peptide might not be the optimal one. Given the non-TPR-mediated function (non-ATP and non-Hsp90) action of Coi12, this has to be considered. Perhaps the authors might want to comment on this in the discussion.

We agree with the referee's comment and have added the following descriptions (underlined) to Result:
"Therefore, although we cannot exclude the possibility that Coi12p has a PPIase activity only for some specific substrate, the FKBDs of Coi12p most likely lack PPIase activity and may instead play a role in protein-protein interactions, similar to many other PPIase-inactive FKBDs (Galat, 2008; Rohl et al., 2013)."

Referee #2:

Major points:

1. Immunoprecipitation is a convenient method to check "binding" between Twi1p and siRNAs. However, it remains unknown if those siRNAs bound to Twi1p in the absence of ATP or the chaperone machinery have really been "loaded" in a canonical manner and produced functional RISC. It is formally possible that, in the absence of ATP or the chaperone machinery, siRNAs are aberrantly bound to Twi1p without efficient RISC formation. Indeed, the in vivo phenotype of Coi12KO + dTPR is extremely severe (Fig. 6), compared to the modest inhibition of siRNA binding

© European Molecular Biology Organization

in the absence of the TPR domain, Hsp90 or ATP in vitro (Fig. 5). The authors should check the double-stranded vs single-stranded states of those Twi1p-coIPed siRNAs in the presence or absence of ATP and show that the two strands are separated in the same kinetics. Measuring and quantitatively comparing their target-slicing activities (normalized to the amount of bound siRNAs) will also be informative.

We examined scnRNAs co-precipitated with Twi1p by a native gel electrophoresis. We used cell lysates from cells expressing FLAG-HA-tagged wild-type Twi1p and FLAG-HA-tagged Twi1p with a Slicer-inactive mutation (D526N, Noto et al. 2010) without ATP addition. We found that RNAs co-precipitated with the wild-type Twi1p were mostly single-stranded whereas RNAs co-precipitated with the Slicer-inactive Twi1p remained partially double-stranded. The results have been added to the revised manuscript as Figure 4D.

Because the immunoprecipitation-based loading assay takes ~1hr to isolate loaded siRNAs, it is not easy to compare kinetics of the loading with and without ATP. Any method analyzing target-slicing activities has not been established for Tetrahymena cell lysate system and we think establishing an assay for this purpose is beyond the scope of this study. However, we agree that this is important point and thus we have added the following sentences to Discussion:

"Because double-stranded scnRNAs loaded into Twi1p were mostly converted into single-stranded RNAs in the cell lysate even without ATP addition (Figure 4D), ATP is dispensable not only for loading but also for passenger strand removal. However, in our loading assay, the loaded scnRNAs were detected by immunoprecipitating Twi1p and thus we most likely observed the only end point of the loading process. Therefore, it is possible that the presence of ATP accelerates passenger strand removal but our assay is not suitable for dissecting the role of ATP in this process."

2. Similarly, the authors should check those siRNAs bound to Twi1p in Gwi1KO lysate (Fig. 7) have produced functional RISC.

We checked the single-strandedness of scnRNAs in the loading assay with *GIW1* KO cell lysate and found that they were mostly single-stranded as in the wild-type cell lysate. Therefore, we believe RNAs are properly loaded to Twi1p in the absence of Giw1p. The result has been added as Fig 7B.

3. It is critical for the authors to validate their "lid model" in vivo. The authors should immunoprecipitate Twi1p from Coi12p and Giw1p double-KO cells and Coi12p single-KO cells, and show that siRNAs are more efficiently loaded into Twi1p in the absence of Giw1p.

For unknown reason, we failed to produce *COI12* and *GIW1* double KO strains. We are not sure whether this is caused by some technical problem or the biological consequence. Because of this problem, we have to leave this question for future studies.

Minor points:

1. The authors should confirm that the same amount of Twi1p was IPed in the presence or absence of ATPgammaS in Fig. 5E, as well as in other in vitro IP experiments.

We have added western blots analyzing IPed Twi1p in Fig. 5A-E and Fig. 7A.

2. The amount of input siRNAs in Fig. 7D should be shown to exclude the possibility that siRNAs were simply degraded by some contaminants in GST-Giw1p.

We incubated siRNAs and GST-Giw1p in the same buffer used for the assay shown in Fig. 7D and found that even the highest concentration (500 nM) of GST-Giw1p used in the assay did not greatly reduce siRNAs. These data have been added as Figure S6 and a description for this control experiment is in Result section.

3. Does Giw1p bind to small RNAs? Is it possible that Twi1p and Giw1p compete for siRNA binding?

© European Molecular Biology Organization

Our preliminary attempts failed to detect the interaction between Giw1p and double-stranded scnRNAs. However, because this is an important and interesting question, we need further efforts to obtain a solid answer for this question. We hope that the referee agrees that this is a topic for future studies.

4. Fig. 7E requires a positive control of wild-type Twi1p, which should predominantly produce ssRNAs.

Recombinantly-expressed Twi1p shows very weak Slicer activity in our hands (Noto et al. 2010) and, probably because of that, a similar experiment with wild-type Twi1p gave an outcome similar to that of Fig 7E. This might be because most of the recombinant Twi1p proteins purified in our hands were Slicer-inactive proteins or because some factor in the cell lysate is important for Twi1p's Slicer activity. Nonetheless, the experiments shown in Fig 7E indicates that scnRNAs are loaded as double-stranded RNAs into recombinant Twi1p. Therefore, we keep the figure as is.

5. Page 17, line 9: Iki et al., 2010, 2012 in fact used tobacco AGO1, not Arabidopsis AGO1.

We have corrected these mistakes in the text.

6. Fig. 5 legend: His-Coi12p-ΔC should read His-Hsp82p-ΔC.

We have corrected these mistakes in the text.

7. The text is sometimes overstated and should be carefully toned down. For example, given the strong in vivo phenotype of Coi12KO+dTPR (Fig. 7), it is too strong to state "Hsp90- and ATP-independent loading of siRNA occurs in vivo and is an integral part of the DNA elimination process in Tetrahymena." Moreover, it remains unknown if their findings are applicable to other species.

We only wanted to claim that Coi12p-ΔTPR has a loading-promoting activity not only in vitro but also in vivo. To make our message clearer without overstating, we have changed some of the descriptions as follows (changed sentences are underlined):

(Summary) "suggesting that Hsp90- and ATP-independent loading of siRNA occurs in vivo and plays a physiological role in Tetrahymena."

(Discussion) "These results indicate that in Tetrahymena, ATP- and Hsp90-independent loading of siRNA duplexes can occur in vivo and that this loading process is not simply a bypass mechanism but ~~an integral part of~~ plays a physiological ~~process~~ role in this organism."

Referee #3:

However, the manuscript could be improved by more thoroughly discussing the relationship between the functions of Giw1p identified in this manuscript and those previously published in Noto, et al. Giw1p has previously been shown to associate preferentially with molecules of Twi1p loaded with single-stranded sRNAs, while the model presented in this manuscript requires Giw1p association with unloaded Twi1p. Reconciling these findings with one another in the discussion would be appreciated.

To better explain the relationship between our previous and present observations, we added the following descriptions (added sentences are underlined). Also, the interaction between unloaded Twi1p and Giw1p was analyzed in *DCL1* KO cells and the data has been added as Figure 7A.

(Results, p20) "We previously showed that Giw1p directly interacts with Twi1p bound by single-stranded but not by double-stranded scnRNA and that this interaction is necessary for the MAC localization of Twi1p (Noto et al. 2010). In the same report, we showed that Giw1p stayed bound to Twi1p even after complete degradation of scnRNAs by RNaseA treatment of wild-type cell lysate, although the biological importance of this interaction was not clear. We confirmed this observation by testing co-precipitation of Twi1p and Giw1p in DCL1 KO cells, in which Twi1p is in an unloaded state due to lack of scnRNAs (Malone et al. 2005; Mochizuki & Gorovsky 2005). The Twi1p-containing complex was immunpprecipitated from wild-type and DCL1 KO cells at 3 hpm

using an anti-Twi1p antibody. Lesser Twi1p was precipitated from DCL1 KO cell than from wild-type cells (Figure 7A, Twi1p, IP) due to the instability of unloaded Twi1p (Figure 3E). Gwi1p was clearly co-precipitated with Twi1p in the absence of DCL1 and the amount of precipitated Gwi1p was correlated with the amount of precipitated Twi1p in wild-type and DCL1 KO cells (Figure 7A, Gwi1p, IP) indicating that Giw1p can interact with unloaded Twi1p in vivo."

(Discussion, p29) "We previously showed that Giw1p binds to Twi1p complexed with single-stranded, but not double-stranded, scnRNAs and that this interaction selectively promotes the MAC localization of the mature Twi1p-scnRNA complex (Noto et al. 2010). In this study, we showed an additional role of Giw1p: this protein interacts with unloaded Twi1p and inhibits loading of small RNAs into Twi1p. It remains unclear how unloaded Twi1p escapes from the MAC import. In addition to its interaction with Giw1p, a conformational change or a post-translational modification of Twi1p that is caused by the loading of scnRNA might be necessary for the MAC import of Twi1p."

2nd Editorial Decision — 08 December 2014

Thank you for submitting your final revised manuscript for our consideration. I am pleased to inform you that we have now accepted it for publication in The EMBO Journal.

Referee #2:

The authors have adequately addressed my previous concerns and the manuscript is now suitable for publication in EMBO J.
